# Lifestyle, Environmental, Occupational, and Dietary Risk Factors in Small-Cell vs. Non-Small-Cell Advanced Lung Cancer Patients: Is There a Connection?

**DOI:** 10.3390/cancers17050864

**Published:** 2025-03-03

**Authors:** Jelena Jovičić-Bata, Danica Sazdanić-Velikić, Mirjana Ševo, Maja Milanović, Teodora Tubić, Milorad Bijelović, Nataša Milošević, Nataša Milić

**Affiliations:** 1 Department of Pharmacy, Faculty of Medicine, University of Novi Sad, 21000 Novi Sad, Serbia; jelena.jovicic-bata@mf.uns.ac.rs (J.J.-B.); maja.milanovic@mf.uns.ac.rs (M.M.); natasa.milic@mf.uns.ac.rs (N.M.); 2 Institute for Pulmonary Diseases of Vojvodina, Clinic for Pulmonary Oncology, Faculty of Medicine, University of Novi Sad, 21204 Sremska Kamenica, Serbia; danica.sazdanic-velikic@mf.uns.ac.rs; 3 Faculty of Medicine, University of Novi Sad, 21000 Novi Sad, Serbia; 902012d23@mf.uns.ac.rs; 4 IMC Banja Luka-Center of Radiotherapy, Part of Affidea Group, 78000 Banja Luka, Bosnia and Herzegovina; 5 Department of Anesthesiology and Perioperative Medicine, Faculty of Medicine, University of Novi Sad, 21000 Novi Sad, Serbia; teodora.tubic@mf.uns.ac.rs; 6 Clinic for Anesthesia, Intensive Care and Pain Therapy, University Clinical Center of Vojvodina, 21000 Novi Sad, Serbia; 7 Institute for Pulmonary Diseases of Vojvodina, Clinic for Thoracic Surgery, 21204 Sremska Kamenica, Serbia; 8 Faculty of Medicine Foča, University of East Sarajevo, 73300 Foča, Bosnia and Herzegovina

**Keywords:** lung cancer, environmental pollution, dietary habits, occupational exposure, lifestyle

## Abstract

Objectives: (i) To evaluate the possible exposure of newly diagnosed lung cancer patients to selected lifestyle, environmental, occupational, and dietary risk factors and (ii) to assess the differences in exposures of small-cell (SCLC) and non-small-cell (NSCLC) lung cancer patients to those risk factors. Methods: In this study, 205 newly diagnosed patients with IIIB/IV stage of either SCLC or NSCLC (111 men vs. 94 women) from Vojvodina, Serbia, were surveyed for selected demographic characteristics, dietary and lifestyle habits, and environmental factors. Results: Most patients were long-term heavy smokers. The body mass index values of SCLC patients were higher than those of NSCLC patients. Women reported higher stress levels compared to men. Women diagnosed with lung adenocarcinoma were more often exposed to traffic pollution compared to men. Individual indoor coal combustion systems were more often used by SCLC patients of both sexes compared to other cancer types. Men were more frequent consumers of canned foods, which are potential sources of endocrine disruptors. Occupational exposure to lung cancer risk factors, in addition to tobacco smoking, may be crucial in lung cancer development with specific occupations. Conclusions: Further research on environmental and occupational risk factors for lung cancer is urgent in order to unveil the etiopathogenesis of specific lung cancer types.

## Simple Summary

Lung cancer remains the leading cause of cancer-related deaths despite significant declines in tobacco-smoking rates. Among non-smokers, lung cancer became the fifth leading cause of cancer-related death, thus putting other modifiable risk factors such as lifestyle, environmental pollution, occupational exposure, and dietary habits in focus. The aim of this study was to evaluate the potential exposure of newly diagnosed patients with advanced lung cancer to selected lifestyle, environmental, occupational, and dietary risk factors and to determine the differences in exposures between small-cell and non-small-cell lung cancer patients. A survey conducted on over 200 lung cancer patients confirmed that, in addition to tobacco smoking as a primary risk factor, environmental pollution, occupational exposure to pollutants, lifestyle, and dietary habits could be identified as modifiable risk factors for lung cancer. Differences in exposure to specific risk factors between genders and types of lung cancer were also observed.

## 1. Introduction

Lung cancer was the most frequently diagnosed cancer worldwide in 2022, accounting for 12.4% of all cancers, as well as the leading cause of cancer deaths, with an estimated 1.8 million deaths (18.7%), according to the latest statistics [1]. Data from Serbia align with global trends, as lung cancers were the overall leading cause of death among all cancer patients in 2021, as well as the leading cause of cancer deaths among men and the second among women, following breast cancer [2]. It is estimated that approximately 15% of all lung cancer cases are classified as small-cell lung cancer (SCLC), while the remaining 85% are grouped as non-small-cell lung cancers, or NSCLCs, such as adenocarcinomas, squamous cell carcinomas, and large cell carcinomas, including large cell neuroendocrine carcinoma.

Recent findings indicate that modifiable risk factors, such as cigarette smoking, obesity/overweight, alcohol consumption, lack of physical activity, diet, and infections, contribute to 4 out of 10 cancer cases and 50% of all cancer deaths in adults over 30 in the USA [3]. Previous tuberculosis infection has been recognized as a risk factor for lung cancer, especially among heavy smokers [4]. Nearly one-fifth of all cancer cases and one-third of all cancer deaths can be attributed to cigarette smoking. However, despite declining global tobacco-smoking rates, lung cancer remains the leading cause of cancer deaths, with observed increases in rates among non-smokers. In 2023, lung cancer in non-smokers became the fifth most common cause of cancer-related deaths [5]. Hence, in clinical evaluations of lung cancer, other risk factors are increasingly coming into focus. Some studies have indicated biological and epidemiological differences in lung cancer in non-smokers, as they are mainly diagnosed with non-small-cell lung cancer, predominantly with adenocarcinoma. The number of non-smoking women with lung cancer appears to be increasing, although their survival times seem longer compared with non-smoking men with lung cancer [6]. The associations between obesity and lung cancer prevalence and outcomes are complex, even though the so-called “obesity paradox” indicates lower rates of carcinoma and better prognoses for patients with higher BMIs. Since the body fat distribution is not clearly described in most studies, the association between central obesity and lung cancer remains unclear [7,8]. Lifestyle factors, including major stressful life events within a period of five years prior to lung cancer diagnosis, are also documented as risk factors [9]. Other everyday life aspects are observed not only as risk factors but also as agents with synergistic effects when combined with smoking that may affect survival rates among lung cancer patients. For example, air pollution is recognized as the second leading cause of lung cancer [10]. Environmental and occupational risk factors include ionizing and non-ionizing radiation, such as radon or ultraviolet (UV) radiation exposure, various chemical substances, including asbestos, metal(loid)s (arsenic, chromium, nickel, cobalt, lead, mercury, etc.), and industrial pollutants, like bisphenols, phthalates, acrylamide, and polycyclic aromatic hydrocarbons, as well as pesticides, like dioxins, chlorimuron-ethyl, and parathion [11,12]. Outdoor air pollution is considered the second leading cause of lung cancer. Construction, industrial emission, transportation, and wildfires are the main sources of outdoor air pollution, which includes both gaseous pollutants and particulate matter. Particulate matter (PM) smaller than 2.5 microns (PM2.5) in diameter is classified as an International Agency for Research on Cancer (IARC) Group 1 carcinogen for humans [10]. Indoor pollution, including cooking practices, household air pollution from solid fuel combustion, and incense burning, was also associated with lung cancer prevalence [10,13,14,15,16]. Although mold was not associated with lung cancer, mycotoxin-linked mutations and lung cancer risk remain in the focus of further investigations [17]. Finally, the protective and harmful effects of specific diets and their link to cancer, and lung cancer specifically, are being continuously monitored [18]. As the diet refers not only to ingredients from foods and beverages, but also to numerous chemicals ingested with meals, due to specific packaging or processing, dietary habits related to the possible incidence of lung cancer require constant caution [19].

Despite all the limitations that observational analysis has, the urgent need for this type of research on lung cancer risk factors, especially environmental ones, contributing to lung cancer in non-smokers and different types of lung cancer is undeniable. Thus, the aim of this study was to evaluate the possible exposure of newly diagnosed lung cancer patients to selected lifestyle, environmental, occupational, and dietary risk factors for lung cancer, as well as to assess the differences in exposures of SCLC and NSCLC patients to those risk factors.

## 2. Materials and Methods

### 2.1. Patients

All patients enrolled in this study were referred to the Institute for Pulmonary diseases of Vojvodina, Sremska Kamenica, Serbia, under the suspicion of lung cancer. They underwent diagnostic procedures, and after pathohistological confirmation of lung cancer, they were surveyed about selected dietary and lifestyle habits, as well as their exposure to environmental risk factors for lung cancer. Patient enrolment was conducted over a two-year period, from March 2022 to February 2024. The inclusion criteria for this study were as follows: age > 18 years, creatinine clearance (CrCl) > 60 mL/min (calculated using the Cockroft–Gault formula), confirmed advanced stage (IIIB or IV) lung cancer, and signed informed consent. Patients with a previous history of lung cancer, those who had received oncological treatment, and pregnant women were excluded from this study. Additional exclusion criteria were any other type of cancer, as well as lung cancer stage < IIIB. Finally, 205 patients with newly diagnosed IIIB/IV stage lung cancer (111 men and 94 women) who provided written informed consent were included in this study.

### 2.2. Research Instrument

A review of the available literature revealed no existing survey that fully addressed our research questions. Therefore, a specific survey on selected demographic characteristics, dietary and lifestyle habits, and environmental factors was developed specifically for this research, based on similar available surveys [20,21,22,23,24]. The questionnaire was evaluated for face and content validity [25]. Further validation was deemed not applicable, as the questionnaire did not measure knowledge or contain psychometric scales (needing Cronbach α, McDonalds ω, or any other parameter determination). The survey used consisted of four sections: demographic characteristics, anthropometric characteristics, clinically relevant information, and a survey on exposures to different lung cancer risks, including some risk factors not specific for lung cancer, but cancers overall. Patients completed the surveys during their scheduled visits to the Institute for Pulmonary Diseases of Vojvodina. Patients unable to complete the surveys independently, due to physical limitations or literacy issues, were assisted by their healthcare providers.

### 2.3. Anthropometric Measurements

BMIs were calculated using clinical measurements of body mass and height, and patients were classified as underweight (<18.50 kg/m^2^), having normal weight (18.50–24.99 kg/m^2^), being overweight (25.00–29.99 kg/m^2^), or obese (≥30.00 kg/m^2^) [26]. Waist and hip circumferences were measured in accordance with the WHO STEPS Surveillance protocol [27] in a clinical setting. Waist circumference (WC) and waist–hip ratio (WHR) cut-off points were chosen in accordance with WHO recommendations for Caucasian populations [26]. WC values associated with increased metabolic risks were defined as >94 cm for men and >80 cm for women. WHRs considered to be high-risk were ≥0.9 for men and ≥0.85 for women [26,28]. Both WC and WHR were categorized as low-risk and high-risk in accordance with the aforementioned cut-off points. Data on waist and hip circumferences were missing for seven and nine patients, respectively. Therefore, it was not possible to calculate WHR for a total of nine patients.

### 2.4. Exposures

Among lifestyle factors, exposure to tobacco products was assessed through a series of questions. Exposure to certain environmental agents and/or pollutants was defined as living or working in close proximity (150 m or less) to an air pollution source for at least a year. Air pollutants were classified according to IARC monographs [29]. Dietary intake was assessed using a semiquantitative food frequency questionnaire, in which participants indicated the frequency of consumption of specific food. For seasonally available foods, intake frequency was reported based on consumption during the respective season. Patients who experienced chronic stress, traumatic life events, daily hassles, and/or acute stress [9,30] within the last five years were identified as exposed to stress.

### 2.5. Statistical Analysis

Categorical variables were described by frequencies. The chi-squared test and, when applicable (expected cell counts < 5), Fisher’s exact test were used for between-group comparisons of categorical variables. The suitability of continuous variables for parametric tests was assessed using QQ plots and Levene’s test. Continuous variables were described using means and standard deviations (SDs), and their differences were assessed using Student’s *t*-test or ANOVA (where applicable) followed by a post hoc Tukey test. Additionally, the frequency distribution of various food consumption patterns was treated as ordinal data; means were used to assess consumption and identify the most frequently consumed foods. An average consumption score was calculated for all fruits combined, as well as for vegetables and saltwater and freshwater fish. Finally, this calculation was performed for each food item individually. A higher average consumption score indicated that the food group or specific food was consumed more frequently than that with a lower average consumption score. Statistical analysis was performed using SPSS Statistics for Windows version 25 (IBM Corporation, Armonk, NY, USA). A *p*-value of <0.05 was considered statistically significant. Percentages in tables do not always sum to 100% due to rounding. The same statistical tests were applied to both the tables presented in the manuscript and those included in the Supplementary Material.

## 3. Results

A total of 205 lung cancer patients were included in the sample (111 males, 94 females; all Caucasian), 60 of whom were diagnosed with SCLC, while 145 were diagnosed with NSCLC. Among NSCLC patients, 66 had lung adenocarcinoma, 62 had squamous cell lung cancer, and 17 were diagnosed with neuroendocrine lung cancer. Sample characteristics including gender, age, and employment status for both SCLC and NSCLC patients are presented in Table 1.

The distribution of men and women was similar across carcinoma types (*p* = 0.195). The mean age of the respondents was 67.8 ± 8.1 years (range 45–86 years) and did not differ by carcinoma type (*p* = 0.098) (Table 1). There were no differences in the employment status (*p* = 0.431) in relation to lung cancer type. Among female patients, 93.6% were menopausal, 28.7% had used oral contraceptives at some point during their reproductive years, 7.4% reported having been prescribed hormonal replacement therapy at some point, and 10.6% had undergone a hysterectomy. No significant differences were observed in these variables between women with SCLC and those with other lung cancer types (Supplementary Material, Table S1). Women reported having 1.8 ± 0.8 children on average, with no significant difference between the SCLC and other carcinoma groups (1.6 ± 0.7 vs. 1.9 ± 0.9, respectively; t = 1.651, *p* = 0.102).

### 3.1. Lifestyle Risk Factors

#### 3.1.1. Tobacco Use

A total of 53.2% of respondents identified as current smokers, 43.9% were ex-smokers, and 2.9% reported never having smoked (Table 2). All enrolled patients who were classified as smokers or ex-smokers smoked cigarettes exclusively. None used other tobacco products, e-cigarettes, or vapes. There were no significant differences in the proportions of smokers, ex-smokers, and non-smokers in relation to the type of cancer respondents had (*p* = 0.882). Analysis by gender (Supplementary Material, Tables S2–S4) showed no statistically significant differences in smoking status by carcinoma type.

The majority of patients grew up or lived in a household in which smoking was present (70.8%). Nearly half of the participants (49.8%) reported being regularly exposed to secondhand smoke at least once per month. No differences were observed in secondhand smoking, growing up, or living in smoking households in relation to lung cancer type.

Respondents had an average smoking history of 38 ± 11 years (range 8–60 years; median 40 years) and smoked 24 ± 10 cigarettes per day (range 3–60; median 20 cigarettes). Therefore, the average smoking exposure was 45.6 pack-years. Among those who had quit smoking, the average time since quitting was 9.24 ± 11.39 years ago (range 0.08–48.00; median 4.00). Fourteen patients reported quitting smoking a month ago, coinciding with their lung cancer diagnosis.

There were no differences in the duration of tobacco smoking between patients with SCLC and NSCLC (*p* = 0.245). However, patients diagnosed with SCLC smoked more cigarettes per day. On average, SCLC patients smoked 27 cigarettes per day (54 pack-years) as opposed to 23 smoked by those diagnosed with other lung cancers (42.55 pack-years) (*p* = 0.047). Among ex-smokers, SCLC patients reported quitting smoking approximately 5 years before their diagnosis, which is significantly shorter than the 10 years reported by NSCLC patients (*p* = 0.038). There were no differences in the proportion of smokers among men and women (*p* = 0.209).

#### 3.1.2. BMI/Obesity

The BMI of SCLC patients (26.1 ± 5.2 kg/m^2^) was statistically significantly higher compared to NSCLC patients (24.5 ± 4.4 kg/m^2^) (*p* = 0.041) as shown in Table 3. However, there was no significant difference in BMI by gender (25.0 ± 4.3 kg/m^2^ for men vs. 24.9 ± 5.2 kg/m^2^ for women, *p* = 0.802). The waist circumference (WC) of SCLC patients was more frequently above the WHO cut-off compared to other lung cancer patients (*p* = 0.018), but this difference was not confirmed when evaluating the waist–hip ratio (WHR) (*p* = 0.497) (Table 3). Men were more likely to be categorized as having a high-risk WHR compared to women (88.8% vs. 58.2%, respectively, *p* < 0.001), while the assessment of WC showed that women were more often categorized as having high-risk WC than men (68.1% vs. 51.4%, respectively, *p* = 0.015).

Further analysis (Supplementary Material, Tables S5–S7) by lung cancer type and gender revealed that, in the NSCLC group, a higher percentage of women were categorized as having high-risk WC compared to men (64.2% vs. 44.9%, *p* = 0.020). Conversely, NSCLC men had high-risk WHR more often than women (89.3% vs. 54.7%, *p* < 0.001). In the SCLC group, men had high-risk WHR more often than women (87.5% vs. 66.7%), but this difference bordered on statistical significance (*p* = 0.054). Among patients with lung adenocarcinoma, the proportion of normal-weight women was higher (though not statistically significant) than that of men (70.4% vs. 51.3%). Similarly, the proportion of obese women with neuroendocrine lung cancer (60.0%) was higher than the respective proportion of men (28.6%), but again, showing no statistically significant difference. The proportion of individuals with low-risk WC was higher in the lung adenocarcinoma group in comparison to groups with other cancer types, whereas the proportion of SCLC patients with high-risk WC was higher than in other lung cancer groups (*p* = 0.011). More specifically, women with neuroendocrine lung cancer had a significantly higher prevalence of high-risk WC compared to men (90.0% vs. 42.9%, *p* = 0.036). Men had high-risk WHR more often than women, both in the lung adenocarcinoma and squamous cell lung cancer group (*p* = 0.028 and *p* = 0.001, respectively).

#### 3.1.3. Stress

No significant differences were observed between SCLC and other lung cancer patients regarding recent (within the last five years) exposure to situations known as stressors, such as illness or death of a close person, relocation, or job loss (SCLC 46.7% vs. NSCLC 55.9%; *p* = 0.230). Stress appeared to be slightly more prevalent among NSCLC patients, though not significantly. However, a more detailed analysis showed that 68.7% of NSCLC women had experienced recent stress compared to 44.9% of men in the same group (*p* = 0.004). Further tests revealed that women with lung adenocarcinoma and neuroendocrine lung cancer were more likely to report stress exposure than men from the same groups (70.4% vs. 35.9%, *p* = 0.006, and 70.0% vs. 14.3%, *p* = 0.024) (Supplementary Material, Tables S8–S10).

### 3.2. Outdoor Pollution

#### 3.2.1. Air Pollution

No differences were observed in the proportions of SCLC patients exposed to air pollution sources compared to those with NSCLC (Table 4). Women with lung adenocarcinoma were more often exposed to traffic pollution compared to men (33.3% vs. 10.3%; *p* = 0.020) (Supplementary Material, Tables S11–S13).

Out of the surveyed lung cancer patients, 42% were drivers, and 16.6% reported driving a diesel car or vehicle (Table 5). No differences were observed between SCLC and other lung cancer patients in response to vehicle type (*p* = 0.863).

The majority of the drivers were men (61.3% men vs. 19.2% women, *p* < 0.001). Gender differences were observed when assessing the types of lung cancer in relation to driving (Supplementary Material, Table S14). More women than men diagnosed with SCLC, as well as other types of lung cancer, were non-drivers (*p* < 0.001 for both categories of cancer) (Supplementary Material, Tables S15–S17).

#### 3.2.2. Electromagnetic Fields

A significant difference was observed among patients with different lung carcinomas in relation to exposure to electromagnetic fields. Namely, SCLC patients were more frequently exposed to electromagnetic pollution compared to NSCLC (SCLC 38.3% vs. NSCLC 21.4%, *p* = 0.012). Overall, the proportion of patients exposed to electromagnetic fields was smaller than that of those who were not exposed. Looking into specific carcinoma types and gender, no differences between exposures to this type of pollution were observed, except for an additional confirmation that SCLC patients were more frequently exposed to electromagnetic fields in comparison to other lung cancer type patients (*p* = 0.035) (Supplementary Material, Tables S18–S20).

### 3.3. Indoor Air Pollution

#### 3.3.1. Household Combustion of Fossil Fuels

SCLC patients were more frequently exposed to pollution from individual heating and cooking combustion systems, typical of rural areas, with individual housing, compared to patients with NSCLC (73.3% vs. 58.6%, *p* = 0.047). Patients diagnosed with SCLC reported using coal as a source of energy for cooking and heating more frequently than other lung cancer patients (15.0% vs. 5.5%, *p* = 0.047). No other differences were observed in the use of other fossil fuels for residential heating and cooking between SCLC and NSCLC patients (Table 6). Further analysis revealed that the use of natural gas was more common in the households of neuroendocrine lung cancer patients than among lung adenocarcinoma patients (58.8% vs. 22.7%, *p* = 0.024), with no other significant differences noted between these groups. SCLC, lung adenocarcinoma, and squamous cell lung cancer patients used wood as a residential heating fuel more frequently than neuroendocrine lung cancer patients (55.0%, 53.0%, 48.4%, respectively, vs. 11.8%, *p* = 0.013). In terms of gender differences, women with squamous cell lung cancer were statistically significantly more often exposed to natural gas combustion in their households compared to men (40.0% vs. 12.5%, *p* = 0.013). Men with squamous cell lung cancer more frequently reported being exposed than women to wood used as fuel for domestic cooking and heating (65.6% vs. 30%, *p* = 0.005) (Supplementary Material, Tables S21–S23). Exposures of men to coal and wood combustion products were notably higher than those of women, although the difference was not statistically significant (11.7% vs. 4.3%, *p* = 0.055 and 55.0% vs. 41.5%, *p* = 0.054, respectively).

#### 3.3.2. Fragrances and Scented Products

Air fresheners were used by 44.9% of our respondents in houses, cars, and offices. Deodorants were used more than three times per week by 42.9%, while perfumes were used daily by 19.5% of the patients. Scented laundry detergent, fabric softeners, and scented household cleaning products were used by 96.1%, 94.6%, and 95.6% of the respondents, respectively. No differences were observed in the proportion of SCLC patients compared to other lung cancer patients in the use of air fresheners, household cleaning products, deodorants, perfumes, and similar scented products. Organic, fragrance-free products were used by 7.3% of respondents (Table 7).

However, men with NSCLC were more often exposed to scented household cleaning products compared to women (98.7% vs. 91.0%, *p* = 0.049), whereas women with NSCLC were more often exposed to organic, fragrance-free cleaning products than men (*p* = 0.007). Deodorant use was more frequent among women than men for both SCLC and NSCLC patients (*p* = 0.006 and *p* < 0.001, respectively) (Supplementary Material, Tables S24–S26).

#### 3.3.3. Household Pesticide Use

Respondents reporting household pesticide use were more often diagnosed with SCLC than other types of lung cancer (SCLC 45.0% vs. NSCLC 22.8%; *p* = 0.001). This difference remained significant when comparing the use of pesticides across all cancer types (*p* = 0.015). Among NSCLC respondents, a greater percentage of men than women reported household pesticide use (35.9% vs. 7.5%, *p* < 0.001). Further analysis showed that men with lung adenocarcinoma and squamous cell lung cancer used pesticides more often than their women counterparts (*p* = 0.008 and *p* = 0.022, respectively) (Supplementary Material, Tables S27–S29).

#### 3.3.4. Mold

The majority of surveyed patients reported not having mold within their households (79.0%). No differences between groups were identified in relation to having mold or dampness present in their households by lung carcinoma type (SCLC 28.3% vs. NSCLC 17.9% *p* = 0.096), nor by specific cancer types or gender (Supplementary Material, Tables S30–S32).

### 3.4. Occupational Exposure

Nearly one-third of the respondents (63, 30.7%) reported occupational exposure to industrial chemicals, primarily industrial paints, lacquers, nitro thinners, and glues. About a quarter of respondents (55, 26.8%) experienced physical problems after being exposed to volatile substances from fragranced products (cleaning products, deodorants, perfumes, hair coloring, and styling products) or paints, smoking or second-hand smoking, exhaust fumes, etc. These issues were most frequently associated with chlorine-based cleaning products which affected 15 individuals (exclusively women) and industrial paints, lacquers, and/or thinners which affected 10 individuals (almost exclusively men). Most of the observed reactions to chlorine-containing products occurred in the neuroendocrine lung cancer group (13/15), whereas paint and lacquers were responsible for 5/10 reactions in the squamous cell lung cancer group.

A total of 22 respondents were professionally linked to agriculture (10 with SCLC, 9 men), 13 were professional drivers (6 with lung adenocarcinoma, all men), 9 worked in plastics and rubber production facilities, and 8 were workers in the textile industry (4 with SCLC, all women). In addition, there were 11 machinists (6 with squamous cell lung carcinoma) and 6 welders (with no apparent dominant cancer type). Apart from these, 27 women were housewives or worked as cleaners.

### 3.5. Dietary Factors

#### 3.5.1. Food Consumption

Fruits had an average consumption score of 3.31 in the SCLC and 3.18 in the NSCLC group, while vegetables had an average consumption score of 3.05 and 3.06 in the SCLC and NSCLC groups, respectively.

The most frequently consumed fruits were apples (average consumption score 3.39), while potatoes (3.82) and tomatoes (3.80) were the most commonly consumed vegetables (Table 8). No differences were noted in fruit and vegetable consumption scores between SCLC and NSCLC groups except for strawberry consumption (*p* = 0.026) (Table 8).

Sardines were the most commonly consumed saltwater fish (average consumption score 1.98), followed by tuna (1.87) and hake (1.71). Carp was the most frequently consumed freshwater fish among the respondents (1.86) (Table 8). No differences in fish consumption frequency were observed between SCLC and NSCLC patients.

When in season, strawberries were more frequently consumed “≥twice per week” by patients with SCLC compared to other lung cancer patients (68.3% vs. 53.1%, *p* = 0.045). Women with NSCLC consumed nectarines “≥twice per week” more often than men did (59.7% vs. 37.2%, *p* = 0.007). Further analysis showed that this difference was primarily observed in women with lung adenocarcinoma (66.7% vs. 41.0%, *p* = 0.040) (Supplementary Material, Tables S33–S36).

#### 3.5.2. Bisphenol A and Polytetrafluoroethylene Exposure

The use of bagged instant soups and canned foods was similar between SCLC and NSCLC groups (*p* = 0.314 and *p* = 0.177, respectively) (Table 9), although men with SCLC were more frequent users of canned foods in comparison to women (*p* = 0.010). The majority of respondents from both SCLC and NSCLC groups reported never using plastic containers for heating foods (*p* = 0.339) (Table 9, Supplementary Material, Tables S37–S39).

No differences in polytetrafluoroethylene-lined cookware use were observed between patients with SCLC and those with other types of lung cancer (SCLC 61.7 vs. NSCLC 65.5%; *p* = 0.600). Further analysis by gender and specific cancer types was also negative for statistical significance (Supplementary Material, Tables S40–S42).

#### 3.5.3. Amalgam Fillings (Mercury—Hg)

Patients with SCLC reported having amalgam dental fillings more frequently than NSCLC patients (SCLC 61.7% vs. NSCLC 45.5%, *p* = 0.035). No differences by gender were observed (Supplementary Material, Tables S43–S45).

## 4. Discussion

This study is the first to assess the exposure of patients with different types of lung cancer to various lung cancer risk factors. In addition, it was also the first to address the exposure of advanced lung cancer patients from Vojvodina, Serbia, to selected environmental, lifestyle, and dietary risk factors for lung cancer. Given that age was recognized as an independent factor for higher mortality risk as well as the stage of lung cancer [31,32], due to physiological changes that occur with aging, including immunosenescence, only patients with advanced stages of lung cancer (IIIB/IV) were included in the survey.

The smoking prevalence in Serbia is on the decline [33,34]; however, the lung cancer incidence rate is on the rise [35]. Currently, Serbia has the second-highest lung cancer prevalence in Europe. Lung cancer was the leading cause of overall cancer-related mortality in Serbia in 2021 [2]. Recent data suggest a high prevalence of lung cancer among women in Serbia [35,36], mirroring the trend observed in the US [37,38]). Therefore, assessing and understanding the patterns of human exposure to lung cancer risk factors, beyond smoking, is crucial for setting up a framework that can be aimed at further reducing the risk of developing lung cancer.

### 4.1. Lifestyle Risk Factors

Tobacco smoking is a major risk for lung cancer, in both active smokers and those exposed to second-hand smoke. While smoking is considered a lifestyle choice, second-hand exposure may reasonably be viewed as an environmental risk factor. The societal burden of tobacco use in Serbia is high [39]. According to the Tobacco Atlas, 38% of Serbian adults (15+ years) are smokers, and tobacco use accounts for 23% of all annual deaths [40]. The vast majority of our respondents were smokers (53.2%) and ex-smokers (43.9%), reinforcing the notion that tobacco smoking is a leading risk factor for lung cancer. In addition, many ex-smokers reported quitting at the time of their diagnoses (14/90). Most of the surveyed patients grew up or lived in households with smokers. In addition, research suggested that living in a smoking household can negatively affect smoking cessation efforts and influence relapse patterns [41]. Our research detected a higher proportion of women tobacco smokers compared to males, although this difference was not statistically significant (*p* = 0.400 for SCLC and *p* = 0.287 for NSCLC, Supplementary Material, Table S2). Among tobacco smokers, women may be at greater risk of developing lung cancer, due to an array of innate characteristics, such as genetic and enzymatic differences from men [42]. Special care should be put into policies aimed towards women smokers as women were historically underrepresented in lung cancer research. Growing evidence suggests that women may have different risk factors, treatment outcomes, and prognoses for lung cancer [42,43]. The high average number of pack-years (45.6) and smoking duration (38 years) among our surveyed patients would be an indication for lung cancer screening. For example, the US Preventive Services Task Force [44] recommends screening for lung cancer in adults aged 50–80 with a 20 pack-year smoking history who have been smokers in the last 15 years. In 2020, a screening program for lung cancer was implemented at the Institute for Pulmonary Diseases of Vojvodina, Sremska Kamenica, Serbia. There are indications that the program will expand to other healthcare centers in Serbia [45,46,47].

Some research suggested a potential inverse association between obesity and lung cancer, often referred to as the “obesity paradox” [8,48,49]. While this phenomenon is likely influenced by confounding factors such as tobacco smoking and its effects on body mass [50], it is possible that BMI influences the risks associated with certain types of lung carcinoma. For example, a higher BMI was linked to an increased risk of squamous cell lung carcinoma [50]. Our results revealed a significantly higher proportion of obese individuals in the SCLC group in comparison to other lung cancer types. The proportion of normal-weight women with lung adenocarcinoma was higher than that of men, and the proportion of obese women with neuroendocrine lung cancer was higher than the respective proportion of men. However, squamous cell lung cancer patients’ BMI did not differ statistically from that of other patients. The noted differences warrant further investigation with a larger sample to better understand the true nature of this relationship.

Research has shown that major stressful events can increase the risk of lung cancer within a five-year period [9]. More recent data [51] implied that emotional distress negatively affects clinical outcomes in advanced-stage NSCLC, but more detailed data one the relationship between specific types of lung cancer and stress remains scarce. Our results supported the fact that recent stress-inducing events affected NSCLC patients more. Additionally, we found that more women diagnosed with NSCLC are being exposed to stress compared to men, specifically those with lung adenocarcinoma and neuroendocrine lung cancer. These gender differences might stem from women being more willing to disclose emotional distress than men are, or this trend is a consequence of different stress-coping mechanisms between genders [52].

### 4.2. Outdoor Pollution

According to the World Health Organization, 99% of the global population lives in areas where air pollution levels do not meet WHO guideline limits [53]. Air pollution is responsible for 6.7 million global deaths per year, primarily affecting people in low- and middle-income countries [54,55]. In Serbia, air pollution was the seventh leading risk factor for deaths in 2019, despite some improvements seen in the 2010s [56]. The electricity production in Serbia is predominantly reliant on lignite power plants, which represent 68% of the total installed capacity of the Serbian Electric Power Industry [57]. In addition, coal mining, especially lignite mining, together with ore mining, mineral processing, and smelting, is a source of PM10 and PM2.5, and in some regions of Serbia, the concentrations of As in PM10 exceeded the limit value, along with slightly elevated cadmium (Cd) values [58]. Moreover, lignite combustion emits twice as much carbon dioxide per unit of energy as natural gas and typically contains more sulfur and ash. Lignite combustion in the power plants in Serbia is not only a significant carbon emission source, but there is a constant increase in the carbon emission factor from the main Serbian power plants [59]. Overall, the majority of our respondents did not live or work in close proximity (<150 m) to air pollution emission sources such as industry, energy production plants and waste management plants, traffic-based pollution, and agricultural areas. This does not necessarily mean they were never exposed to these emission sources but rather suggests a more limited amount of exposure. In addition, this might be attributed to the fact that most of the major industrial sources of air pollutants are located in central Serbia/outside of Vojvodina [60]. Conversely, the Vojvodina region is regularly affected by stubble burning, a common practice in cultivated fields [61]. This practice even led to the launching of a portal for reporting and monitoring fires on agricultural lands [62]. Stubble burning releases a variety of pollutants including carbon dioxide (CO_2_), carbon monoxide (CO), nitrogen oxides (NO_x_), sulfur oxides (SO_x_), and methane (CH_4_), as well as particulate matter (PM10 and PM2.5) [63].

All aforementioned gaseous pollutants and particulate matter have been linked to lung cancer [64,65,66,67]. Certain air pollutants’ concentrations might be correlated to a higher incidence of specific lung cancer subtypes. A Polish study [68] showed a specifically significant effect of SO_2_ on lung adenocarcinoma incidence in women. A Taiwanese study [69] suggested that exposure to air pollutants such as NO_2_, often associated with traffic pollution, increases lung adenocarcinoma rates. Although our study did not distinguish between specific air pollutants, we did find that women, more frequently exposed to traffic pollution, had a higher likelihood of being diagnosed with lung adenocarcinoma than men. In Serbia, levels of SO_2_ and NO_2_ fluctuated seasonally [70], but more recent research showed falling trends in SO_2_ concentrations [71]. In neighboring Bosnia and Herzegovina, elevated NO_2_ concentrations in the urban city center of Bijeljina have been recorded [72].

Diesel exhaust has been linked to increased lung cancer risks [73,74,75] and is classified as a Group 1 human carcinogen. Although research [76,77] showed an association between diesel exhaust exposure and increased risk for squamous cell lung carcinoma, our data did not reveal a similar relationship.

A substantial body of data suggests that pesticide exposure from agricultural activities increases the risk of lung cancer [78,79,80,81]. However, most of these studies were focused on populations with occupational exposure to pesticides. Recently, an epidemiological analysis [82] concluded that living in areas with higher agricultural activity raises the risks for all cancers, including lung cancer. Data on specific lung cancer types associated with non-occupational pesticide exposure are scarce. Our data showed no difference between the proportions of SCLC and NSCLC patients living close to agricultural areas.

The IARC classified radiofrequency electromagnetic fields (EMFs) as possible human carcinogens (Group 2B) in 2011 [83], citing an increased risk for glioma associated with wireless phone use. On the other hand, the WHO [84], the European Commission [85], the US National Toxicology Program [86], and the FDA [21,87] reported no clear evidence linking EMF exposure to an increase in cancer risk. However, some studies indicated an increase in cancer mortality with EMF exposure [88]. Our results suggested that among those exposed to EMF, SCLC was a dominant lung cancer subtype. Data on the EMF effects and risks for specific lung cancer subtypes are not available. Therefore, our findings might warrant a more in-depth investigation.

### 4.3. Indoor Air Pollution

Household combustion of fossil fuels emits carbon monoxide, nitrous oxides, particulate matter (PM2.5, PM10), and/or a variety of air toxins like polycyclic aromatic hydrocarbons (PAHs) and volatile organic compounds (VOCs), all of which can cause health problems [89]. It is important to recognize that this exposure might be seasonal, depending on the heating and cooking household routines. Household combustion of coal is classified as a Group 1 carcinogen [90], whereas biomass fuel consisting mostly of wood is classified as Group 2A [91]. Solid fossil fuel combustion for heating and cooking increases lung cancer risk, proportionate to the duration of exposure [92]. As previously mentioned, most of our patients were exposed to individual fossil fuel combustion systems used for residential heating and cooking (62.9%), with wood-burning systems being the most common (a total of 48.8% of those using fossil fuels). This exposure rate is similar to that of the US general population (two-thirds), but US citizens mainly use cleaner energy sources, like natural gas (60%) [93]. Apart from other pollutants, wood burning emits PAHs which significantly increase lung cancer risk in enclosed spaces such as homes with open fireplaces or poorly ventilated rooms [94,95] since PAHs are classified as Group 1 (benzo[a]pyrene), Group 2A, and Group 2B [96]. Wood burning for cooking has been shown to increase lung cancer risk among non-smoking women to a lesser extent than coal use [97] as coal combustion releases more toxic substances. In Serbia, over 40 million tons of coal is produced annually, with lignite of lower calorific value being the most abundant fossil fuel used in thermal power plants and occasionally in household heating systems [98]. Gender differences observed in our results stem from the traditional roles of women, as they are the ones who typically prepare food within the household [42] and are, therefore, exposed to these pollutants for a longer time. Our research showed no differences in exposure to different fossil fuel combustion systems. That said, given the borderline *p*-values for wood burning (*p* = 0.054) and coal use (*p* = 0.055), a larger sample size (drawn from the same stage IIIB/IV lung cancer patient pool) might reveal a significant relationship. Nevertheless, it is worth noting that simple methods like opening windows, creating air draft, etc., might considerably decrease the risks associated with fossil fuel combustion, especially in the case of its use for cooking, and should, therefore, be promoted.

Limited data are available on the patterns of fossil fuel use in relation to lung cancer subtypes. Specifically, PM2.5 exposure has been linked to a higher incidence of squamous cell lung cancers [99] and lung adenocarcinoma [66]. In our research, neuroendocrine lung cancer patients more often used natural gas for heating compared to patients with SCLC, squamous cell lung cancer, or lung adenocarcinoma. Patients who used wood were more often diagnosed with SCLC, squamous cell lung cancer, or lung adenocarcinoma.

Volatile organic compounds (VOCs) can be inhaled from scented personal care products, cleaning products, air fresheners, and other products intended to evaporate scent. A single “scent” can be made up of up to 300 specific ingredients [100], but can be labeled simply as “fragrance”, providing no further information about ingredients. Such a lack of transparency leaves consumers unaware of their exposure to VOCs. Otherwise, scented products contribute to indoor air pollution and can cause adverse health effects ranging from irritation to an increased risk of developing lung cancer, some directly by being carcinogenic, others by increasing susceptibility to lung cancer [101,102]. In our study, almost all participants used some scented product. Deodorant use was more frequent among women than men, both in SCLC and other carcinoma groups, but this finding probably reflects women’s care and hygiene habits [103]. Among our participants, more men with NSCLC were exposed to cleaning products compared to women. Given the traditional gender roles, this may indicate occupational exposure rather than household use. Alternatively, it could suggest underreporting by women due to routine product use [104,105]. We noted no differences in exposures to scented products in relation to specific lung cancer types. However, Chen et al. [13] observed an increased risk of lung adenocarcinoma associated with incense burning. In addition, scented products for personal use and cosmetics are known as a source of phthalates, endocrine disruptors with possible links to hormone-dependent cancers of particular importance for women [106].

Our survey demonstrated differences in household exposure to pesticides among patients diagnosed with different lung cancer types. However, data on this issue are scarce as most research has focused primarily on occupational exposure to pesticides (mentioned previously). Kangkhetkron et al. [78] demonstrated an increase in lung cancer risks in persons exposed to specific pesticides (non-occupational) but did not differentiate between lung cancer types. As the link between non-occupational exposure to pesticides and lung cancer remains unclear, it is possible that our findings are coincidental. Nevertheless, overexposure to pesticides is still possible when pesticides are not used in accordance with their labels.

Molds are responsible for many health problems, but there is no evidence supporting the direct association between exposure to mold and lung cancer [107]. Our research supports this finding by presenting no difference in exposure to mold or dampness among different lung cancer groups.

### 4.4. Occupational Exposure

Unfortunately, occupational exposure to certain agents may increase the risk of developing lung cancer. The IARC’s list of carcinogenic and possibly carcinogenic agents for lung cancer in humans [67] identifies several agents specifically as occupational risk factors. Miners, professional drivers, toll-booth workers, and construction workers are at risk of developing lung cancer due to their exposure to diesel exhausts (IARC Group 1) [75,108]. Animal studies suggested an increased risk for lung adenocarcinoma and squamous cell lung carcinoma in diesel-exhaust-exposed rats [86] which corresponded to our findings—a total of 13 of our respondents were drivers, with half of them suffering from lung adenocarcinoma. Although currently authorized pesticides are required to be non-genotoxic [109], (chronic) exposure to high levels of some pesticides, such as occupational exposure, increases lung cancer risks [11,79,80,81,110,111]. Improper or unauthorized handling and use of pesticides might further increase these risks. Among our respondents, 22 reported working in agriculture, with half of them diagnosed with SCLC. Garcia et al. [111] showed that occupational exposure to pesticides is a risk factor for lung cancer, specifically SCLC. The results obtained in this study are concerning, knowing that our patients might have been exposed to a variety of pesticides that were banned in Serbia just in mid-November 2023 [112].

The rubber manufacturing process [113], as well as acrylonitrile used in plastics and resin production, has been identified by the IARC as carcinogenic with no specific mention of any lung cancer type. There were nine workers from these industries among our participants, but with no obvious dominance of any lung cancer type. Welders (a total of six of our respondents) have been reported to be at higher risk of lung cancer. This might be due to exposure to welding fumes classified as IARC Group 1 [114] carcinogens and, in part, possibly due to exposure to inorganic lead (IARC Group 2A) [115]. In particular, welders are at higher risk of developing SCLC and squamous cell lung cancer [116], but we detected no dominance of any lung cancer type among welders in this study.

### 4.5. Dietary Risk Factors

An important issue to consider is the contamination of food with potential or proven carcinogens. Foods that are often contaminated with cadmium, like vegetables (especially starchy vegetables) [117], might increase cancer risks. Namely, apples, potatoes, and tomatoes, some of the most frequently consumed foods by patients included in this study, have been shown to have high lead and/or cadmium content [118]. Green leafy vegetables, consumed frequently by more than half of the participants in this study, were found to have cadmium and lead levels exceeding FAO/WHO maximum permissible levels in certain areas [119,120]. In a study conducted in Serbia [120], nearly 20% of the analyzed samples of leafy vegetables had cadmium and almost 10% had lead levels above the permissible levels. Although lead was not often present in quantifiable amounts in lung cancer patients in Vojvodina, it is still important to monitor the populations’ exposure to lead, as lead compounds have been classified as IARC Group 2A (inorganic compounds) and Group 3 (organic compounds) [121]. However, high concentrations of cadmium and mercury have already been detected in urine samples of lung cancer patients from Vojvodina [122]. Since our study implied potatoes as the most frequently consumed vegetable, this might be considered a potential exposure route for cadmium. Mercury might enter the human food chain through fat fish, such as tuna and sardines [123], which were the most frequently consumed fish. Furthermore, the intake of canned fish (sardines, tuna) might also be linked to bisphenol A exposure (see below) because of bisphenol A’s tendency to migrate from can linings to foods that are high in fats [124]. High-fat foods may also be a source of dioxins and dioxin-like substances, some of which are classified as Group 1 by the IARC [125].

Novakov et al. [126] found that levels of Cd, As, Fe, and benzo(a)pyrene in samples of canned saltwater fish available on the Serbian market exceeded both the European and Serbian regulatory limits. However, Popović et al. [127] reported that the levels of As, Cd, Pb, and Hg in canned fish presented a low risk to the Serbian population, considering current consumption trends. Sabo et al. [128] noted the absence of pesticides in fish (and other animals), along with the presence of Cd (above LOQ), Pb, Hg, As, and Mn (below LOQ) in fish samples from canals and ditches around Bački Petrovac in Serbia. It is worth noting that these results were limited to a specific location and that in the majority of sampling locations on major water flows in Serbia located downstream from wastewater outlets of public utility companies, the quality of surface water is considered to be low/bad according to the Serbian Water Quality Index (SWQI) [129]. The practice of unregulated sales of the catch originating from illegal, unreported, and unregulated fishing at local marketplaces raises concerns about the exposure of the general population to wastewater residues and contaminants.

In addition, not discussed in this paper is the issue of other proven and possible carcinogens like inorganic and organic arsenic compounds. The ingestion of arsenic through drinking water has been estimated to be as high as 60 μg per day [130] in the region of Vojvodina. Previously, the presence of arsenic in lung cancer patients in Vojvodina has been reported [122].

Bisphenol A (BPA) is an endocrine disruptor present in plastic materials, food containers, and the inner coatings of food containers and bags that can leach into foods [131]. Although the data on the link between BPA and cancer are conflicting [132,133,134], there are indications that BPA can increase lung cancer risk [135]. Our research noted almost no differences in the exposure of lung cancer patients to BPA from foods, except in the proportion of men and women with SCLC exposed to BPA through instant canned food. This difference could be attributed to gender-based dietary habits, as men have a preference to consume convenient, ready-to-eat foods over cooking from scratch [136,137,138]. Moreover, heating food in plastic containers might present an additional risk due to BPA and phthalate leakage into foods, but the correlation between such practice and cancer is still not thoroughly validated [131,139].

Per- and polyfluoroalkyl substances (PFASs) are often used in coatings of non-stick cookware and food packaging. Despite the IARC classifying some PFAS members as Group 1 and Group 2B carcinogens [140], no direct link to lung cancer was found. Additionally, the use of PFASs as polymers limits their potential to migrate into foods and cause adverse effects. Damaged coatings and food packaging increase the PFAS migration risk into foods. The potential increase in cancer risks associated with the use of non-stick cookware might be stimulated by frequent exposure to cooking oil fumes affecting cancer risk [141], and not necessarily by the cookware itself. However, our research did not detect any differences in exposures of different lung cancer type patients to non-stick cookware coatings. Men with SCLC seemed to be more frequent users of non-stick cookware products (72.7%) in comparison to women with SCLC (48.1%), but this difference was borderline significant (*p* = 0.051).

Mercury has been implicated in lung carcinogenesis [142,143], but no link between lung cancer or specific lung cancer types and amalgam dental fillings has been proven. In our study, SCLC patients reported having amalgam fillings more frequently than NSCLC patients, but these differences are unlikely to be attributable to mercury leaching. Namely, urinary levels of mercury in lung cancer patients in Vojvodina were not associated with the presence of dental fillings [122]. The US and EU recommend against the use of amalgam dental fillings in vulnerable population groups due to possible mercury toxicity [144,145]. Additionally, the European Union has announced a ban on amalgam fillings, effective from 2025, due to concerns regarding toxicity and environmental impact [146].

### 4.6. Strengths and Limitations

The main advantage of this research is that it is the first to assess the variety of exposures of different newly diagnosed advanced lung cancer patients to selected lung cancer risk factors. Similar smoking habits of all surveyed patients are consistent with the exclusion of smoking as the only risk factor affecting our patients. Lastly, as the observation of exposures was performed shortly after diagnosis, patients had no time to correct and/or change most of their behaviors/jobs prior to being surveyed. Thus, the reported exposures accurately reflect patients’ habits. Considering the above, our research offers a glimpse into the patterns of lung cancer patients’ exposure to selected cancer risk factors from a unique, multifactor perspective.

The main limitation of this research is its monocentric setup, as well as the homogeneity of the sample. Therefore, the results are difficult to generalize across different ethnicities and geographical areas. This research, as a descriptive observational study, cannot support any causal relationship, but can only generate hypotheses on the cause of the pathology itself. Another limitation consists in the risk of “false positivity” present in the “multiplicity” of significance tests carried out on the same sample, since each comparison is not carried out “with all other variables being equal”, due to the failure to neutralize all known and unknown confounding factors. The failure to introduce an effect threshold can be noted [147], and finally, as in every survey, bias is possible, as is the underreporting of undesirable behavior and vice versa. A larger sample obtained from the same stage IIIB/IV lung cancer patient pool would enhance the statistical power of the results, making their extrapolation to the population more reliable. The evaluation of the lifestyle, environmental, occupational, and dietary risk factors among different stages and types of lung cancer would require a significantly larger sample size and a multicenter approach.

## 5. Conclusions

In this study, we may assume that various risk factors could influence the incidence of different lung cancer types. Most of the patients were long-term heavy smokers, laying the groundwork for lung cancer from an early age. Taking into account the unfavorable epidemiological data on lung cancer in Serbia, resources should be directed towards the promotion of different mitigation strategies for lung cancer risk factors, including education about smoking’s adverse health effects. The BMI of SCLC patients was higher than that of NSCLC patients, but further investigation should be performed to determine the nature of these relationships. Women reported higher stress levels compared to men, possibly making them more vulnerable to lung cancer. Women who were more often exposed to traffic pollution had a higher likelihood of being diagnosed with lung adenocarcinoma than men. SCLC patients of both sexes were more frequently exposed to individual, indoor combustion systems, especially coal combustion systems. The common use of low-calorific coal, lignite, may be the most important environmental lung cancer risk factor in Serbia. A high proportion of wood use was noted among SCLC, lung adenocarcinoma, and squamous cell lung cancer in comparison to neuroendocrine lung cancer patients. Some gender differences in fossil fuel use by cancer type were also noted. The differences in the use of fragranced products were mainly gender-related, with little implications of the link between them and lung cancer. Our findings revealed men were more frequent consumers of canned foods, potential sources of endocrine disruptors, which should be monitored as probable dietary risk factors for lung cancer. Better control of environmental risk factors could result in lower levels of some contaminants in foods and water and pave the way to achieving better health within a healthier environment. Finally, besides tobacco smoking, occupational exposure to lung cancer risk factors can play a pivotal role in lung cancer development in specific occupations. More research is warranted to help us understand the relationships between different risk factors for lung cancer and their potential to contribute to the etiopathogenesis of specific lung cancer types.

## Figures and Tables

**Table 1:** Gender, age, and employment status of lung cancer patients with SCLC and NSCLC enrolled in this study.

		SCLC		NSCLC				
		N/x	%/SD	N/x	%/SD	*p*	Total	%/SD
Gender	male	33	55.0	78	53.8	0.195	111	54.1
	female	27	45.0	67	46.2		94	45.8
Age		66.2	8.9	68.3	7.8	0.098	67.8	8.1
Employment status	employed	13	21.7	31	21.4	0.431	44	21.5
	retired	35	58.3	95	65.5		130	63.4
	unemployed	12	20.0	19	13.1		31	15.1

**Table 2:** Patients’ exposure to tobacco by lung cancer types.

		SCLC		NSCLC				
		N	%	N	%	*p*	Total	%
Smoking status	current smoker	33	55.0	76	52.4	0.882	109	53.2
	ex-smoker	26	43.3	64	44.1		90	43.9
	non-smoker	1	1.7	5	3.4		6	2.9
Passive smoking	multiple times/month	30	50.0	72	49.7	0.964	102	49.8
	1× per month or less	30	50.0	73	50.3		103	50.2
Smoking household *	yes	46	78.0	97	67.8	0.150	143	69.8

* Household members’ smoking status was missing for three participants.

**Table 3:** Patients’ anthropometric characteristics by lung cancer type.

		SCLC		NSCLC				
		N/x	%/SD	N/x	%/SD	*p*	Total	%/SD
BMI		26.1	5.2	24.5	4.4	0.041	25.0	4.7
BMI	underweight	2	3.3	8	5.5	0.097	10	4.9
	normal	31	51.7	79	54.5		110	53.7
	overweight	11	18.3	39	26.9		50	24.4
	obese	16	26.7	19	13.1		35	17.1
WC *	low-risk	17	28.3	67	46.2	0.018	84	41.0
	high-risk	43	71.7	78	53.8		121	59.0
WHR *	low-risk	13	22.0	37	26.6	0.497	50	24.4
	high-risk	46	78.0	102	73.4		148	72.2

* Data on waist and hip circumferences were missing for seven and nine patients, respectively; WC—waist circumference; WHR—waist–hip ratio.

**Table 4:** Proportions of participants exposed to air pollution sources by cancer type.

	SCLC		NSCLC			Total	
	N	%	N	%	*p*	N	%
Industrial air pollution (incl. factories and waste management systems)	4	6.7	8	5.5	0.750	12	5.8
Energy production facilities (incl. heating plants, power plants, and refineries)	4	6.7	11	7.6	0.818	15	7.3
Waste management facilities (incl. landfills, incineration stations)	4	6.7	3	2.1	0.099	7	3.4
Transportation-generated pollution (incl. highways, gas stations, parking facilities)	16	26.7	26	17.9	0.159	42	20.5
Agricultural areas	23	38.3	45	31.0	0.313	68	33.2

**Table 5:** Proportions of patients exposed to diesel exhaust by cancer type.

		SCLC		NSCLC			Total	
		N	%	N	%	*p*	N	%
Diesel car/vehicle	yes	11	18.3	23	15.9	0.863	34	16.6
	no	14	23.3	38	26.2		52	25.4
	not a driver	35	58.3	84	57.9		119	58.0

**Table 6:** Proportion of patients exposed to selected fossil fuel combustion systems by cancer type.

	SCLC		NSCLC			Total	
	N	%	N	%	*p*	N	%
Individual heating/cooking combustion systems	44	73.3	85	58.6	0.047	129	62.9
Natural gas	15	25.0	41	28.3	0.632	56	27.3
Coal	9	15.0	8	5.5	0.047	17	8.3
Wood	33	55.0	67	46.2	0.252	100	48.8
Oil	1	1.7	0	0.0	-	1	0.5

**Table 7:** Proportion of patients exposed to fragrances and scented products by lung cancer type.

		SCLC		NSCLC			Total	
		N	%	N	%	*p*	N	%
Air fresheners		21	35.0	71	49.0	0.067	92	44.9
Scented household cleaning products		58	96.7	138	95.2	0.531	196	95.6
Organic, fragrance-free cleaning products		3	5.0	12	8.3	0.580	15	7.3
Deodorants	3+ times per week	22	36.7	66	45.5	0.244	88	42.9
	less than 2 times/week	38	63.3	79	54.5		117	57.1
Perfumes, colognes, etc.	everyday	9	15.0	31	21.4	0.294	40	19.5
	not everyday	51	85.0	114	78.6		165	80.5
Scented laundry detergent		58	96.7	139	95.9	0.787	197	96.1
Fabric softener		59	98.3	135	93.1	0.131	194	94.6

**Table 8:** Mean consumption scores of selected fruits, vegetables, and fishes by lung cancer type.

	SCLC		NSCLC		
	x	SD	x	SD	*p*
Apples	3.52	0.892	3.33	1.00	0.214
Grapes	3.17	1.181	3.31	1.017	0.412
Apricots	3.50	0.873	3.27	1.082	0.111
Peaches	3.42	0.979	3.33	1.007	0.577
Nectarines	2.90	1.217	2.81	1.280	0.657
Strawberries	3.38	1.027	3.01	1.228	0.026
Cucumbers	3.60	0.827	3.46	0.921	0.316
Celery	2.62	0.993	2.62	1.068	0.980
Cherry tomatoes	2.17	1.224	2.16	1.305	0.967
Peas	2.55	0.790	2.50	0.826	0.670
Spinach	2.40	0.887	2.47	0.928	0.625
Tomatoes	3.78	0.666	3.81	0.601	0.749
Potatoes	3.82	0.504	3.82	0.467	0.956
Peppers	3.80	0.514	3.63	0.762	0.073
Green, leafy vegetables	3.27	1.039	3.26	0.970	0.942
Tuna	1.87	0.853	1.88	0.927	0.947
Salmon	1.10	0.440	1.05	0.245	0.394
Sardine	2.02	0.911	1.96	0.904	0.677
Mackerel	1.37	0.681	1.37	0.697	0.955
Hake	1.58	0.743	1.77	0.882	0.161
Herring	1.02	0.129	1.05	0.272	0.391
Catfish	1.48	0.748	0.41	0.584	0.477
Carp	1.85	0.820	1.87	0.757	0.874
Sterlet	1.02	0.129	1.03	0.183	0.493

**Table 9:** Proportion of participants exposed to BPA via its selected sources.

	SCLC		NSCLC			Total	
	N	%	N	%	*p*	N	%
Bagged instant soups	29	48.3	59	40.7	0.314	88	42.9
Canned foods	31	51.7	60	41.4	0.177	91	44.4
Heating up foods in plastic containers	5	8.3	7	4.8	0.331	12	5.8

## Data Availability

The datasets generated during and/or analyzed during the current study are not publicly available due to personal data protection, but are available from the corresponding author on reasonable request.

## Supplementary Materials

The following supporting information can be downloaded at https://www.mdpi.com/article/10.3390/cancers17050864/s1: Table S1: Specific characteristics regarding reproductive health of the female lung cancer patients enrolled in this study; Table S2: Smoking status of the enrolled patients with SCLC and NSCLC based on their gender (given as proportions of the exposed respondents); Table S3: Smoking status of the enrolled lung cancer patients based on the diagnosed cancer type (given as proportions of the exposed respondents); Table S4: Smoking status of the enrolled lung cancer patients based on their gender and the diagnosed cancer type (given as proportions of the exposed respondents); Table S5: Anthropometric characteristics of the enrolled patients with SCLC and NSCLC based on their gender (given as proportions of the exposed respondents); Table S6: Anthropometric characteristics of the enrolled lung cancer patients based on the diagnosed cancer type (given as proportions of the exposed respondents); Table S7: Anthropometric characteristics of the enrolled lung cancer patients based on the diagnosed cancer type and their gender (given as proportions of the exposed respondents); Table S8: Exposure to recent stress of the enrolled patients with SCLC and NSCLC based on their gender (given as proportions of the exposed respondents); Table S9: Exposure to recent stress of the enrolled lung cancer patients based on the diagnosed cancer type (given as proportions of the exposed respondents); Table S10: Exposure to recent stress of the enrolled lung cancer patients based on the diagnosed cancer type and their gender (given as proportions of the exposed respondents); Table S11: Exposure to air pollution of the enrolled patients with SCLC and NSCLC based on their gender (given as proportions of the exposed respondents); Table S12: Exposure to air pollution of the enrolled lung cancer patients based on the diagnosed cancer type (given as proportions of the exposed respondents); Table S13: Exposure to air pollution of the enrolled lung cancer patients according to the diagnosed cancer type and their gender (given as proportions of the exposed respondents); Table S14: Exposure to diesel exhausts of the enrolled lung cancer patients according to their gender (given as proportions of the exposed respondents); Table S15: Exposure to diesel exhausts of the enrolled patients with SCLC and NSCLC according to their gender (given as proportions of the exposed respondents) Table S16: Exposure to diesel exhausts of the enrolled lung cancer patients according to the diagnosed cancer type (given as proportions of the exposed respondents); Table S17: Exposure to diesel exhausts of the enrolled lung cancer patients according to the diagnosed cancer type and their gender (given as proportions of the exposed respondents); Table S18: Exposure to electromagnetic fields of the enrolled patients with SCLC and NSCLC according to their gender (given as proportions of the exposed respondents); Table S19: Exposure to electromagnetic fields of the enrolled lung cancer patients according to the diagnosed cancer type (given as proportions of the exposed respondents); Table S20: Exposure to electromagnetic fields of the enrolled lung cancer patients according to the diagnosed cancer type and their gender (given as proportions of the exposed respondents); Table S21: Exposure to household combustion of fossil fuels of the enrolled patients with SCLC and NSCLC according to their gender (given as proportions of the exposed respondents); Table S22: Exposure to household combustion of fossil fuels of the enrolled lung cancer patients according to the diagnosed cancer type (given as proportions of the exposed respondents); Table S23: Exposure to household combustion of fossil fuels of the enrolled lung cancer patients according to the diagnosed cancer type and their gender (given as proportions of the exposed respondents); Table S24: Exposure to fragrances and scented products of the enrolled patients with SCLC and NSCLC according to their gender (given as proportions of the exposed respondents); Table S25: Exposure to fragrances and scented products of the enrolled lung cancer patients according to the diagnosed cancer type (given as proportions of the exposed respondents); Table S26: Exposure to fragrances and scented products of the enrolled lung cancer patients according to the diagnosed cancer type and their gender (given as proportions of the exposed respondents); Table S27: Use of household pesticides reported by the enrolled patients with SCLC and NSCLC based on their gender (given as proportions of the exposed respondents); Table S28: Use of household pesticides reported by the enrolled lung cancer patients based on the diagnosed cancer type (given as proportions of the exposed respondents); Table S29: Use of household pesticides reported by the enrolled lung cancer patients based on the diagnosed cancer type and their gender (given as proportions of the exposed respondents); Table S30: Exposure to mold of the enrolled patients with SCLC and NSCLC according to their gender (given as proportions of the exposed respondents); Table S31: Exposure to mold of the enrolled lung cancer patients according to the diagnosed cancer type (given as proportions of the exposed respondents); Table S32: Exposure to mold of the enrolled lung cancer patients according to the diagnosed cancer type and their gender (given as proportions of the exposed respondents); Table S33: Proportions of the enrolled lung cancer patients consuming selected foods two times per week or more by the diagnosed lung cancer type; Table S34: Consumption of selected fruits, vegetables, and fishes at least twice a week of patients with SCLC and NSCLC according to their gender (given as proportions of the exposed respondents); Table S35: Consumption of selected fruits, vegetables, and fishes at least twice a week reported by the enrolled lung cancer patients based on the diagnosed cancer type (given as proportions of the exposed respondents); Table S36: Consumption of selected fruits, vegetables, and fishes at least twice a week reported by the enrolled lung cancer patients based on the diagnosed cancer type and their gender (given as proportions of the exposed respondents); Table S37: Exposure to BPA through different sources of the enrolled patients with SCLC and NSCLC based on their gender (given as proportions of the exposed respondents); Table S38: Exposure to BPA through different sources of the enrolled lung cancer patients based on the diagnosed cancer type (given as proportions of the exposed respondents); Table S39: Exposure to BPA through different sources of the enrolled lung cancer patients according to the diagnosed cancer type and their gender (given as proportions of the exposed respondents); Table S40: Exposure to polytetrafluoroethylene of the enrolled patients with SCLC and NSCLC according to their gender (given as proportions of the exposed respondents); Table S41: Exposure to polytetrafluoroethylene of the enrolled lung cancer patients based on the diagnosed cancer type (given as proportions of the exposed respondents); Table S42: Exposure to polytetrafluoroethylene of the enrolled lung cancer patients according to the diagnosed cancer type and their gender (given as proportions of the exposed respondents); Table S43: Proportions of the enrolled patients with SCLC and NSCLC who reported having amalgam dental fillings based on their gender; Table S44: Proportions of the enrolled lung cancer patients with amalgam dental fillings according to the diagnosed cancer type; Table S45. Proportions of the enrolled lung cancer patients with amalgam dental fillings according to the diagnosed cancer type and their gender.

## Abbreviations

The following abbreviations are used in this manuscript:

SCLC	Small-cell lung cancer
NSCLC	Non-small-cell lung cancer
CrCL	Creatinine clearance
BMI	Body mass index
WHR	Waist–hip ratio
WC	Waist circumference
WHO	World Health Organization
FDA	Food and Drug Administration
PAH	Polycyclic aromatic hydrocarbons
EMF	Electromagnetic fields
VOC	Volatile organic compounds
PM	Particulate matter
LOQ	Limit of quantification
BPA	Bisphenol A
PFAS	Per- and polyfluoroalkyl substance
IARC	International Agency for Research on Cancer
SWQI	Serbian Water Quality Index
