# Supplementary material for: Lifestyle, Environmental, Occupational, and Dietary Risk Factors in Small-Cell vs. Non-Small-Cell Advanced Lung Cancer Patients: Is There a Connection?
Source: Cancers (Basel). 2025 Mar 3;17(5):864. doi: 10.3390/cancers17050864 (PMC11899463; doi:10.3390/cancers17050864)
Supplement: Supplementary file 1 [file cancers-17-00864-s001.zip › cancers-3462347-supplementary.pdf]

Lifestyle, Environmental, Occupational, and Dietary Risk Factors in Small-Cell vs. Non-Small-Cell Advanced Lung Cancer Patients: Is There a Connection?

Jelena Jovičić-Bata [1], Danica Sazdanić-Velikić [2], Mirjana Ševo [3,4], Maja Milanović [1], Teodora Tubić [5,6], Milorad Bijelović [7,8], Nataša Milošević [1,*] and Nataša Milić [1]

Correspondence: author: Nataša Milošević, natasa.milosevic@mf.uns.ac.rs

Table S1. Specific characteristics regarding reproductive health of the female lung cancer patients enrolled in the study

	SCLC*		NSCLC**				
	N/x	%/SD	N/x	%/SD	p	Total	%/SD
Menopausal	25	92.6	63	94.0	0.796	88	93.6
Amenorrhea	3	11.1	3	4.5	0.234	6	6.4
Use of oral contraceptives	9	33.3	18	26.9	0.531	27	28.7
Histerectomy	0[1]	0.0	10	14.9	0.058	10	10.6
Hormone replacement therapy	2	7.4	5	7.5	0.993	7	7.4
Number of children	1.6	0.7	1.9	0.9	0.102	1.8	0.8

[1] This category is not used in comparisons because its column proportion is equal to zero or one.
*SCLC – small-cell lung cancer
**NSCLC – non-small-cell lung cancer

Table S2. Smoking status of the enrolled patients with SCLC and NSCLC based on their gender (given as proportions of the exposed respondents)

		SCLC					NSCLC				
		Males		Females			Males		Females		
		N	%	N	%	p	N	%	N	%	p
Smoking status	Smoker	16	48.5	17	63.0	0.400	37	47.4	39	58.2	0.287
	Ex-smoker	16	48.5	10	37.0		39	50.0	25	37.3	
	Non- smoker	1	3.0	0[1]	0.0		2	2.6	3	4.5	

[1] This category is not used in comparisons because its column proportion is equal to zero or one.

Table S3. Smoking status of the enrolled lung cancer patients based on the diagnosed cancer type (given as proportions of the exposed respondents)

		SCLC		Lung adenocarcinoma		Squamous cell lung cancer		Neuroendocrine lung cancer		
		N	%	N	%	N	%	N	%	p
Smoking status	Smoker	33	55.0	29	43.9	36	58.1	11	64.7	0.504
	Ex-smoker	26	43.3	33	50.0	25	40.3	6	35.3	
	Non- smoker	1	1.7	4	6.1	1	1.6	0[1]	0.0	

[1] This category is not used in comparisons because its column proportion is equal to zero or one.

Table S4. Smoking status of the enrolled lung cancer patients based on their gender and the diagnosed cancer type (given as proportions of the exposed respondents)

		SCLC					Lung adenocarcinoma					Squamous cell lung cancer					Neuroendocrine lung cancer				
		Males		Females			Males		Females			Males		Females			Males		Females		
		N	%	N	%	p	N	%	N	%	p	N	%	N	%	p	N	%	N	%	p
Smoking status	Smoker	16	48.5	17	63.0	0.400	15	38.5	14	51.9	0.456	18	56.3	18	60.0	0.523	4	57.1	7	70.0	0.585
	Ex-smoker	16	48.5	10	37.0		22	56.4	11	40.7		14	43.8	11	36.7		3	42.9	3	30.0	
	Non- smoker	1	3.0	0[1]	0.0		2	5.1	2	7.4		0[1]	0.0	1	3.3		0[1]	0.0	0[1]	0.0	

[1] This category is not used in comparisons because its column proportion is equal to zero or one.

Table S5. Anthropometric characteristics of the enrolled patients with SCLC and NSCLC based on their gender (given as proportions of the exposed respondents)

		SCLC					NSCLC				
		Males		Females			Males		Females		
		N	%	N	%	p	N	%	N	%	p
BMI[1]	Underweight	1	3.0	1	3.7	0.916	4	5.1	4	6.0	0.980
	Normal weight	17	51.5	14	51.9		42	53.8	37	55.2	
	Overweight	7	21.2	4	14.8		21	26.9	18	26.9	
	Obese	8	24.2	8	29.6		11	14.1	8	11.9	
WC*[2]	Low-risk	11	34.4	6	22.2	0.304	43	57.3	24	37.5	0.020
	High-risk	21	65.6	21	77.8		32	42.7	40	62.5	
WHR*[3]	Low-risk	4	12.5	9	33.3	0.054	8	10.7	29	45.3	<0.001
	High-risk	28	87.5	18	66.7		67	89.3	35	54.7	

*Data on waist and hip circumferences were missing for seven and nine patients, respectively

[1]BMI – body mass index

[2]WC – waist circumference
[3]WHR – waist-hip ratio

Table S6. Anthropometric characteristics of the enrolled lung cancer patients based on the diagnosed cancer type (given as proportions of the exposed respondents)

		SCLC		Lung adenocarcinoma		Squamous cell lung cancer		Neuroendocrine lung cancer		
		N	%	N	%	N	%	N	%	p
BMI	Underweight	2	3.3	3	4.5	5	8.1	0[1]	0.0	0.158
	Normal weight	31	51.7	39	59.1	33	53.2	7	41.2	
	Overweight	11	18.3	17	25.8	14	22.6	8	47.1	
	Obese	16	26.7	7	10.6	10	16.1	2	11.8	
WC*	Low–risk	17	28.8	37	57.8	25	42.4	5	31.3	0.009
	High-risk	42	71.2	27	42.2	34	57.6	11	68.8	
WHR*	Low–risk	13	22.4	17	27.0	13	22.0	7	43.8	0.315
	High-risk	45	77.6	46	73.0	46	78.0	9	56.3	

*Data on waist and hip circumferences were missing for seven and nine patients, respectively
[1] This category is not used in comparisons because its column proportion is equal to zero or one.

Table S7. Anthropometric characteristics of the enrolled lung cancer patients based on the diagnosed cancer type and their gender (given as proportions of the exposed respondents)

		SCLC					Lung adenocarcinoma					Squamous cell lung cancer					Neuroendocrine lung cancer				
		Males		Females			Males		Females			Males		Females			Males		Females		
		N	%	N	%	p	N	%	N	%	p	N	%	N	%	p	N	%	N	%	p
BMI	Underweight	1	3.0	1	3.7	0.916	3	7.7	0	0.0	0.261	1	3.1	4	13.3	0.492	0[1]	0.0	0[1]	0.0	0.435
	Normal weight	17	51.5	14	51.9		20	51.3	19	70.4		18	56.3	15	50.0		4	57.1	3	30.0	
	Overweight	7	21.2	4	14.8		12	30.8	5	18.5		7	21.9	7	23.3		2	28.6	6	60.0	
	Obese	8	24.2	8	29.6		4	10.3	3	11.1		6	18.8	4	13.3		1	14.3	1	10.0	
WC*	Low-risk	11	34.4	6	22.2	0.304	24	64.9	13	48.1	0.181	15	48.4	10	35.7	0.325	4	57.1	1	11.1	0.049
	High–risk	21	65.6	21	77.8		13	35.1	14	51.9		16	51.6	18	64.3		3	42.9	8	88.9	

WHR*	Low–risk	4	12.9	9	33.3	0.063	6	16.2	11	42.3	0.022	0	0.0	13	46.4	0.001	2	28.6	5	55.6	0.280
	High-risk	27	87.1	18	66.7		31	83.8	15	57.7		31	100.0	15	53.6		5	71.4	4	44.4	

*Data on waist and hip circumferences were missing for seven and nine patients, respectively

[1] This category is not used in comparisons because its column proportion is equal to zero or one.

Table S8. Exposure to recent stress of the enrolled patients with SCLC and NSCLC based on their gender (given as proportions of the exposed respondents)

	SCLC					NSCLC				
	Males		Females			Males		Females		
	N	%	N	%	p	N	%	N	%	p
Exposure to recent stress	14	42.4	14	51.9	0.466	35	44.9	46	68.7	0.004

Table S9. Exposure to recent stress of the enrolled lung cancer patients based on the diagnosed cancer type (given as proportions of the exposed respondents)

	SCLC		Lung adenocarcinoma		Squamous cell lung cancer		Neuroendocrine lung cancer		
	N	%	N	%	N	%	N	%	p
Exposure to recent stress	28	46.7	33	50.0	40	64.5	8	47.1	0.191

Table S10. Exposure to recent stress of the enrolled lung cancer patients based on the diagnosed cancer type and their gender (given as proportions of the exposed respondents)

	SCLC					Lung adenocarcinoma					Squamous cell lung cancer					Neuroendocrine lung cancer				
	Males		Females			Males		Females			Males		Females			Males		Females		
	N	%	N	%	p	N	%	N	%	p	N	%	N	%	p	N	%	N	%	p
Exposure to recent stress	14	42.4	14	51.9	0.466	14	35.9	19	70.4	0.006	20	62.5	20	66.7	0.732	1	14.3	7	70	0.024

Table S11. Exposure to air pollution of the enrolled patients with SCLC and NSCLC based on their gender (given as proportions of the exposed respondents)

	SCLC					NSCLC				
	Males		Females			Males		Females		
	N	%	N	%	p	N	%	N	%	p
Industrial air pollution (incl. factories, and waste management systems)	1	3.0	3	11.1	0.212	6	7.7	2	3.0	0.216
Energy production facilities (incl. heating plants, power plants and refineries)	1	3.0	3	11.1	0.212	7	9.0	4	6.0	0.496
Waste management facilities (incl. landfills, incineration stations)	1	3.0	3	11.1	0.212	3	3.8	0[1]	0.0	0.105
Transportation-generated pollution (incl. highways, gas stations, parking facilities)	9	27.3	7	25.9	0.907	10	12.8	16	23.9	0.083

Agricultural areas	10	30.3	13	48.1	0.157	20	25.6	25	37.3	0.130

[1] This category is not used in comparisons because its column proportion is equal to zero or one.

Table S12. Exposure to air pollution of the enrolled lung cancer patients based on the diagnosed cancer type (given as proportions of the exposed respondents)

	SCLC		Lung adenocarcinoma		Squamous cell lung cancer		Neuroendocrine lung cancer		
	N	%	N	%	N	%	N	%	p
Industrial air pollution (incl. factories, and waste management systems)	4	6.7	3	4.5	4	6.5	1	5.9	0.944
Energy production facilities (incl. heating plants, power plants and refineries)	4	6.7	3	4.5	7	11.3	1	5.9	0.552
Waste management facilities (incl. landfills, incineration stations)	4	6.7	2	3.0	0[1]	0.0	1	5.9	0.124
Transportation-generated pollution (incl. highways, gas stations, parking facilities)	16	26.7	13	19.7	7	11.3	6	35.3	0.074
Agricultural areas	23	38.3	17	25.8	23	37.1	5	29.4	0.408

[1] This category is not used in comparisons because its column proportion is equal to zero or one.

Table S13. Exposure to air pollution of the enrolled lung cancer patients according to the diagnosed cancer type and their gender (given as proportions of the exposed respondents)

	SCLC					Lung adenocarcinoma					Squamous cell lung cancer					Neuroendocrine lung cancer				
	Males		Females			Males		Females			Males		Females			Males		Females		
	N	%	N	%	p	N	%	N	%	p	N	%	N	%	p	N	%	N	%	p
Industrial air pollution (incl. factories, and waste management systems)	1	3.0	3	11.1	0.318	3	7.7	0[1]	0.0	0.264	2	6.3	2	6.7	0.947	1	14.3	0[1]	0.0	0.412
Energy production facilities (incl. heating plants, power plants and refineries)	1	3.0	3	11.1	0.318	1	2.6	2	7.4	0.509	6	18.8	1	3.3	0.055	0[1]	0.0	1	10.0	0.412
Waste management facilities (incl. landfills, incineration stations)	1	3.0	3	11.1	0.318	2	5.1	0[1]	0.0	0.509	0[1]	0.0	0[1]	0.0	NA	1	14.3	0[1]	0.0	0.412
Transportation-generated pollution (incl. highways, gas stations, parking facilities)	9	27.3	7	25.9	0.907	4	10.3	9	33.3	0.020	4	12.5	3	10.0	0.756	2	28.6	4	40.0	0.627
Agricultural areas	10	30.3	13	48.1	0.157	8	20.5	9	33.3	0.242	10	31.3	13	43.3	0.325	2	28.6	3	30.0	0.949

NA – not applicable

[1] This category is not used in comparisons because its column proportion is equal to zero or one.

Table S14. Exposure to diesel exhausts of the enrolled lung cancer patients according to their gender (given as proportions of the exposed respondents)

		Males		Females		
		N	%	N	%	p
Diesel car/vehicle	Yes	31	27.9	3	3.2	<0.001
	No	37	33.3	15	16.0	
	Not a driver	43	38.7	76	80.9	

Table S15. Exposure to diesel exhausts of the enrolled patients with SCLC and NSCLC according to their gender (given as proportions of the exposed respondents)

		SCLC					NSCLC				
		Males		Females			Males		Females		
		N	%	N	%	p	N	%	N	%	p
Diesel car/vehicle	Yes	11	33.3	0[1]	0.0	<0.001	20	25.6	3	4.5	<0.001
	No	10	30.3	4	14.8		27	34.6	11	16.4	
	Not a driver	12	36.4	23	85.2		31	39.7	53	79.1	

[1] This category is not used in comparisons because its column proportion is equal to zero or one.

Table S16. Exposure to diesel exhausts of the enrolled lung cancer patients according to the diagnosed cancer type (given as proportions of the exposed respondents)

		SCLC		Lung adenocarcinoma		Squamous cell lung cancer		Neuroendocrine lung cancer		
		N	%	N	%	N	%	N	%	p
Diesel car/vehicle	Yes	11	18.3	13	19.7	6	9.7	4	23.5	0.513
	No	14	23.3	17	25.8	15	24.2	6	35.3	
	Not a driver	35	58.3	36	54.5	41	66.1	7	41.2	

Table S17. Exposure to diesel exhausts of the enrolled lung cancer patients according to the diagnosed cancer type and their gender (given as proportions of the exposed respondents)

		SCLC					Lung adenocarcinoma					Squamous cell lung cancer					Neuroendocrine lung cancer				
		Males		Females			Males		Females			Males		Females			Males		Females		
		N	%	N	%	p	N	%	N	%	p	N	%	N	%	p	N	%	N	%	p
Diesel car/vehicle	Yes	11	33.3	0[1]	0.0	<0.001	12	30.8	1	3.7	0.001	5	15.6	1	3.3	0.020	3	42.9	1	10.0	0.124
	No	10	30.3	4	14.8		13	33.3	4	14.8		11	34.4	4	13.3		3	42.9	3	30.0	
	Not a driver	12	36.4	23	85.2		14	35.9	22	81.5		16	50.0	25	83.3		1	14.3	6	60.0	

[1] This category is not used in comparisons because its column proportion is equal to zero or one.

Table S18. Exposure to electromagnetic fields of the enrolled patients with SCLC and NSCLC according to their gender (given as proportions of the exposed respondents)

	SCLC					NSCLC				
	Males		Females			Males		Females		
	N	%	N	%	p	N	%	N	%	p
Electromagnetic fields	13	39.4	10	37.0	0.852	13	16.7	18	26.9	0.135

Table S19. Exposure to electromagnetic fields of the enrolled lung cancer patients according to the diagnosed cancer type (given as proportions of the exposed respondents)

	SCLC		Lung adenocarcinoma		Squamous cell lung cancer		Neuroendocrine lung cancer		
	N	%	N	%	N	%	N	%	p
Electromagnetic fields	23	38.3	12	18.2	17	27.4	2	11.8	0.035

Table S20. Exposure of the electromagnetic fields of the enrolled lung cancer patients according to the diagnosed cancer type and their gender (given as proportions of the exposed respondents)

	SCLC					Lung adenocarcinoma					Squamous cell lung cancer					Neuroendocrine lung cancer				
	Males		Females			Males		Females			Males		Females			Males		Females		
	N	%	N	%	p	N	%	N	%	p	N	%	N	%	p	N	%	N	%	p
Electromagnetic fields	13	39.4	10	37.0	0.852	4	10.3	8	29.6	0.057	9	28.1	8	26.7	0.898	0[1]	0.0	2	20.0	0.208

[1] This category is not used in comparisons because its column proportion is equal to zero or one.

Table S21. Exposure to household combustion of fossil fuels of the enrolled patients with SCLC and NSCLC according to their gender (given as proportions of the exposed respondents)

	SCLC					NSCLC				
	Males		Females			Males		Females		
	N	%	N	%	p	N	%	N	%	p
Individual heating/cooking combustion systems	25	75.8	19	70.4	0.639	47	60.3	38	56.7	0.666
Natural gas	11	33.3	4	14.8	0.099	18	23.1	23	34.3	0.134
Coal	6	18.2	3	11.1	0.445	7	9.0	1	1.5	0.049
Wood	18	54.5	15	55.6	0.938	43	55.1	24	35.8	0.020
Oil	1	3.0	0[1]	0.0	0.362	0[1]	0.0	0[1]	0.0	NA

[1] This category is not used in comparisons because its column proportion is equal to zero or one.

Table S22. Exposure to household combustion of fossil fuels of the enrolled lung cancer patients according to the diagnosed cancer type (given as proportions of the exposed respondents)

	SCLC		Lung adenocarcinoma		Squamous cell lung cancer		Neuroendocrine lung cancer		
	N	%	N	%	N	%	N	%	p
Individual heating/cooking combustion systems	44	73.3	37	56.1	38	61.3	10	58.8	0.230
Natural gas	15	25.0	15	22.7	16	25.8	10	58.8	0.024
Coal	9	15.0	5	7.6	3	4.8	0[1]	0.0	0.107
Wood	33	55.0	35	53.0	30	48.4	2	11.8	0.013
Oil	1	1.7	0[1]	0.0	0[1]	0.0	0[1]	0.0	0.376

[1] This category is not used in comparisons because its column proportion is equal to zero or one.

Table S23. Exposure to household combustion of fossil fuels of the enrolled lung cancer patients according to the diagnosed cancer type and their gender (given as proportions of the exposed respondents)

	SCLC					Lung adenocarcinoma					Squamous cell lung cancer					Neuroendocrine lung cancer				
	Males		Females			Males		Females			Males		Females			Males		Females		
	N	%	N	%	p	N	%	N	%	p	N	%	N	%	p	N	%	N	%	p
Individual heating/cooking combustion systems	25	75.8	19	70.4	0.639	22	56.4	15	55.6	0.945	19	59.4	19	63.3	0.749	6	85.7	4	40.0	0.059
Natural gas	11	33.3	4	14.8	0.099	9	23.1	6	22.2	0.935	4	12.5	12	40.0	0.013	5	71.4	5	50.0	0.622
Coal	6	18.2	3	11.1	0.495	4	10.3	1	3.7	0.641	3	9.4	01	0.0	0.238	0[1]	0.0	0[1]	0.0	NA
Wood	18	54.5	15	55.6	0.938	21	53.8	14	51.9	0.873	21	65.6	9	30.0	0.005	1	14.3	1	10.0	0.787
Oil	1	3.0	0[1]	0.0	0.362	0[1]	0.0	0[1]	0.0	NA	0[1]	0.0	0[1]	0.0	NA	0[1]	0.0	0[1]	0.0	NA

[1] This category is not used in comparisons because its column proportions is equal to zero or one.

Table S24. Exposure to fragrances and scented products of the enrolled patients with SCLC and NSCLC according to their gender (given as proportions of the exposed respondents)

		SCLC					NSCLC				
		Males		Females			Males		Females		
		N	%	N	%	p	N	%	N	%	p
Air fresheners		12	36.4	9	33.3	0.807	39	50.0	32	47.8	0.788
Scented household cleaning products		32	97.0	26	96.3	0.885	77	98.7	61	91.0	0.049
Organic, fragrance-free cleaning products		1	3.0	2	7.4	0.583	2	2.6	10	14.9	0.007
Deodorants	yes, every day to couple of times per week	7	21.2	15	55.6	0.006	23	29.5	43	64.2	<0.001
	not at all or <2-3× per week	26	78.8	12	44.4		55	70.5	24	35.8	
Perfumes, colognes, etc.	yes, everyday	2	6.1	7	25.9	0.397	13	16.7	18	26.9	0.135
	not at all or not everyday	31	93.9	20	74.1		65	83.3	49	73.1	
Scented laundry detergent		33[1]	100.0	25	92.6	0.198	74	94.9	65	97.0	0.686
Fabric softener		33[1]	100.0	26	96.3	0.450	73	93.6	62	92.5	0.803

[1] This category is not used in comparisons because its column proportion is equal to zero or one.

Table S25. Exposure to fragrances and scented products of the enrolled lung cancer patients according to the diagnosed cancer type (given as proportions of the exposed respondents)

	SCLC	Lung adenocarcinoma	Squamous cell lung cancer	Neuroendocrine lung cancer

		N	%	N	%	N	%	N	%	p
Air fresheners		21	35.0	36	54.5	26	41.9	9	52.9	0.137
Scented household cleaning products		58	96.7	64	97.0	57	91.9	17[1]	100.0	0.531
Organic, fragrance-free cleaning products		3	5.0	4	6.1	6	9.7	2	11.8	0.580
Deodorants	yes. every day to couple of times per week	22	36.7	31	47.0	28	45.2	7	41.2	0.671
	not at all or <2-3× per week	38	63.3	35	53.0	34	54.8	10	58.8	
Perfumes, colognes, etc.	yes, everyday	9	15.0	15	22.7	13	21.0	3	17.6	0.721
	not at all or not everyday	51	85.0	51	77.3	49	79.0	14	82.4	
Scented laundry detergent		58	96.7	63	95.5	59	95.2	17[1]	100.0	0.098
Fabric softener		59	98.3	64	97.0	55	88.7	16	94.1	0.098

[1] This category is not used in comparisons because its column proportion is equal to zero or one.

Table S26. Exposure to fragrances and scented products of the enrolled lung cancer patients according to the diagnosed cancer type and their gender (given as proportions of the exposed respondents)

	SCLC					Lung adenocarcinoma					Squamous cell lung cancer					Neuroendocrine lung cancer				
	Males		Females			Males		Females			Males		Females			Males		Females		
	N	%	N	%	p	N	%	N	%	p	N	%	N	%	p	N	%	N	%	p
Air fresheners	12	36.4	9	33.3	0.807	22	56.4	14	51.9	0.715	14	43.8	12	40.0	0.765	3	42.9	6	60.0	0.637

Scented household cleaning products		32	97.0	26	96.3	0.885	39[1]	100.0	25	92.6	0.164	31	96.9	26	86.7	0.189	7[1]	100.0	10[1]	100.0	NA
Organic, fragrance-free cleaning products		1	3.0	2	7.4	0.583	1	2.6	3	11.1	0.297	1	3.1	5	16.7	0.099	0[1]	0.0	2	20.0	0.485
Deodorants	yes, every day to couple of times per week	7	21.2	15	55.6	0.006	14	35.9	17	63.0	0.030	9	28.1	19	63.3	0.005	0[1]	0.0	7	70.0	0.010
	not at all or <2-3× per week	26	78.8	12	44.4		25	64.1	10	37.0		23	71.9	11	36.7		7[1]	100.0	3	30.0	
Perfumes, colognes, etc.	yes, everyday	2	6.1	7	25.9	0.397	8	20.5	7	25.9	0.606	5	15.6	8	26.7	0.286	0[1]	0.0	3	30.0	0.110
	not at all or not everyday	31	93.9	20	74.1		31	79.5	20	74.1		27	84.4	22	73.3		7[1]	100.0	7	70.0	
Scented laundry detergent		33[1]	100.0	25	92.6	0.198	37	94.9	26	96.3	0.785	30	93.8	29	96.7	0.593	7[1]	100.0	10[1]	100.0	NA
Fabric softener		33[1]	100.0	26	96.3	0.450	38	97.4	26	96.3	0.791	28	87.5	27	90.0	0.756	7[1]	100.0	9	90.0	0.388

[1] This category is not used in comparisons because its column proportion is equal to zero or one.

Table S27. Use of household pesticides reported by the enrolled patients with SCLC and NSCLC based on their gender (given as proportions of the exposed respondents)

	SCLC					NSCLC				
	Males		Females			Males		Females		
	N	%	N	%	p	N	%	N	%	p
At-home use of pesticides	18	54.5	9	33.3	0.100	28	35.9	5	7.5	<0.001

Table S28. Use of household pesticides reported by the enrolled lung cancer patients based on the diagnosed cancer type (given as proportions of the exposed respondents)

	SCLC		Lung adenocarcinoma		Squamous cell lung cancer		Neuroendocrine lung cancer		
	N	%	N	%	N	%	N	%	p
At-home use of pesticides	27	45.0	16	24.2	14	22.6	3	17.6	0.015

Table S29. Use of household pesticides reported by the enrolled lung cancer patients based on the diagnosed cancer type and their gender (given as proportions of the exposed respondents)

	SCLC					Lung adenocarcinoma					Squamous cell lung cancer					Neuroendocrine lung cancer				
	Males		Females			Males		Females			Males		Females			Males		Females		
	N	%	N	%	p	N	%	N	%	p	N	%	N	%	p	N	%	N	%	p
At-home use of pesticides	18	54.5	9	33.3	0.100	14	35.9	2	7.4	0.008	11	34.4	3	10.0	0.022	3	42.9	0[1]	0.0	0.051

[1] This category is not used in comparisons because its column proportion is equal to zero or one.

Table S30. Exposure to mold of the enrolled patients with SCLC and NSCLC according to their gender (given as proportions of the exposed respondents)

	SCLC					NSCLC				
	Males		Females			Males		Females		
	N	%	N	%	p	N	%	N	%	p
Mold	7	21.2	10	37.0	0.176	11	14.1	15	22.4	0.195

Table S31. Exposure to mold of the enrolled lung cancer patients according to the diagnosed cancer type (given as proportions of the exposed respondents)

	SCLC		Lung adenocarcinoma		Squamous cell lung cancer		Neuroendocrine lung cancer		
	N	%	N	%	N	%	N	%	p
Mold	17	28.3	15	22.7	10	16.1	1	5.9	0.151

Table S32. Exposure to mold of the enrolled lung cancer patients according to the diagnosed cancer type and their gender (given as proportions of the exposed respondents)

	SCLC					Lung adenocarcinoma					Squamous cell lung cancer					Neuroendocrine lung cancer				
	Males		Females			Males		Females			Males		Females			Males		Females		
	N	%	N	%	p	N	%	N	%	p	N	%	N	%	p	N	%	N	%	p
Mold	7	21.2	10	37.0	0.176	6	15.4	9	33.3	0.087	4	12.5	6	20.0	0.422	1	14.3	0[1]	0.0	0.412

[1] This category is not used in comparisons because its column proportion is equal to zero or one.

Table S33. Proportions of the enrolled lung cancer patients consuming selected foods two times per week or more by the diagnosed lung cancer type

	SCLC		NSCLC		
	N	%	N	%	p
FRUITS AND VEGETABLES					
Apples	44	73.3	92	63.4	0.173
Grapes	37	61.7	89	61.4	0.969
Apricots	42	70.0	91	62.8	0.323

Peaches	41	68.3	91	62.8	0.448
Nectarines	28	46.7	69	47.6	0.904
Strawberries	41	68.3	77	53.1	0.045
Cucumbers	46	76.7	101	69.7	0.311
Celery	11	18.3	32	22.1	0.550
Cherry tomatoes	14	23.3	42	29.0	0.410
Peas	7	11.7	13	9.0	0.553
Spinach	8	13.3	21	14.5	0.830
Tomatoes	53	88.3	130	89.7	0.781
Potatoes	52	86.7	124	85.5	0.830
Peppers	51	85.0	113	77.9	0.250
Green, leafy vegetables	35	58.3	78	53.8	0.552
FISH					
Tuna	4	6.7	8	5.5	0.750
Salmon	1	1.7	0[1]	0.0	0.119
Sardine	6	10.0	8	5.5	0.247
Mackerel	6	10.0	8	5.5	0.262
Hake	1	1.7	7	4.8	0.288
Herring	0[1]	0.0	0[1]	0.0	NA
Catfish	2	3.3	0[1]	0.0	0.257
Carp	3	5.0	5	3.4	0.602
Sterlet	0[1]	0.0	0[1]	0.0	NA

[1]This category is not used in comparisons because its column proportion is equal to zero or one.

Table S34. Consumption of selected fruits, vegetables and fishes at least twice a week of patients with SCLC and NSCLC according to their gender (given as proportions of the exposed respondents)

	SCLC					NSCLC				
	Males		Females			Males		Females		
	N	%	N	%	p	N	%	N	%	p
Apples	26	78.8	18	66.7	0.291	46	59.0	46	68.7	0.227
Grapes	20	60.6	17	63.0	0.852	47	60.3	42	62.7	0.764
Apricots	24	72.7	18	66.7	0.610	48	61.5	43	64.2	0.743

Peaches	24	72.7	17	63.0	0.419	47	60.3	44	65.7	0.501
Nectarines	15	45.5	13	48.1	0.835	29	37.2	40	59.7	0.007
Strawberries	21	63.6	20	74.1	0.387	40	51.3	37	55.2	0.635
Cucumbers	25	75.8	21	77.8	0.854	54	69.2	47	70.1	0.905
Celery	5	15.2	6	22.2	0.481	13	16.7	19	28.4	0.091
Cherry tomatoes	7	21.2	7	25.9	0.668	23	29.5	19	28.4	0.881
Peas	3	9.1	4	14.8	0.492	9	11.5	4	6.0	0.242
Spinach	4	12.1	4	14.8	0.760	9	11.5	12	17.9	0.277
Tomatoes	29	87.9	24	88.9	0.903	70	89.7	60	89.6	0.970
Potatoes	30	90.9	22	81.5	0.285	66	84.6	58	86.6	0.739
Peppers	27	81.8	24	88.9	0.445	56	71.8	57	85.1	0.055
Green, leafy vegetables	19	57.6	16	59.3	0.895	38	48.7	40	59.7	0.186
Tuna	3	9.1	1	3.7	0.405	4	5.1	4	6.0	0.825
Salmon	0[1]	0.0	1	3.7	0.265	0[1]	0.0	0[1]	0.0	NA
Sardine	5	15.2	1	3.7	0.141	6	7.7	2	3.0	0.216
Mackerel	0[a]	0.0	0[a]	0.0	NA	1	1.3	2	3.0	0.473
Hake	1	3.0	0[a]	0.0	0.362	2	2.6	5	7.5	0.170
Herring	0[a]	0.0	0[a]	0.0	NA	0[a]	0.0	0[a]	0.0	NA
Catfish	0[a]	0.0	2	7.4	0.112	0[a]	0.0	0[a]	0.0	NA
Carp	1	3.0	2	7.4	0.439	2	2.6	3	4.5	0.529
Sterlet	0[a]	0.0	0[a]	0.0	NA	0[a]	0.0	0[a]	0.0	NA

[1] This category is not used in comparisons because its column proportion is equal to zero or one.

Table S35. Consumption of selected fruits, vegetables and fishes at least twice a week reported by the enrolled lung cancer patients based on the diagnosed cancer type (given as proportions of the exposed respondents)

	SCLC		Lung adenocarcinoma		Squamous cell lung cancer		Neuroendocrine lung cancer		
	N	%	N	%	N	%	N	%	p
Apples	44	73.3	38	57.6	39	62.9	15	88.2	0.056
Grapes	37	61.7	46	69.7	32	51.6	11	64.7	0.212
Apricots	42	70.0	46	69.7	33	53.2	12	70.6	0.151
Peaches	41	68.3	42	63.6	37	59.7	12	70.6	0.727
Nectarines	28	46.7	34	51.5	24	38.7	11	64.7	0.223

Strawberries	41	68.3	33	50.0	32	51.6	12	70.6	0.091
Cucumbers	46	76.7	46	69.7	43	69.4	12	70.6	0.792
Celery	11	18.3	10	15.2	16	25.8	6	35.3	0.205
Cherry tomatoes	14	23.3	20	30.3	16	25.8	6	35.3	0.707
Peas	7	11.7	9	13.6	2	3.2	2	11.8	0.216
Spinach	8	13.3	9	13.6	9	14.5	3	17.6	0.973
Tomatoes	53	88.3	57	86.4	56	90.3	17[1]	100.0	0.432
Potatoes	52	86.7	55	83.3	53	85.5	16	94.1	0.719
Peppers	51	85.0	49	74.2	49	79.0	15	88.2	0.382
Green, leafy vegetables	35	58.3	33	50.0	33	53.2	12	70.6	0.433
Tuna	4	6.7	6	9.1	2	3.2	0[1]	0.0	0.367
Salmon	1	1.7	0[1]	0.0	0[1]	0.0	0[1]	0.0	0.488
Sardine	6	10.0	5	7.6	2	3.2	1	5.9	0.513
Mackerel	0[1]	0.0	1	1.5	1	1.6	1	5.9	0.361
Hake	1	1.7	2	3.0	4	6.5	1	5.9	0.535
Herring	0[1]	0.0	0[1]	0.0	0[1]	0.0	0[1]	0.0	NA
Catfish	2	3.3	0[1]	0.0	0[1]	0.0	0[1]	0.0	0.881
Carp	3	5.0	2	3.0	2	3.2	1	5.9	0.901
Sterlet	0[1]	0.0	0[1]	0.0	0[1]	0.0	0[1]	0.0	NA

[1] This category is not used in comparisons because its column proportion is equal to zero or one.

Table S36. Consumption of selected fruits, vegetables and fishes at least twice a week reported by the enrolled lung cancer patients based on the diagnosed cancer type and their gender (given as proportions of the exposed respondents)

	SCLC					Lung adenocarcinoma					Squamous cell lung cancer					Neuroendocrine lung cancer				
	Males		Females			Males		Females			Males		Females			Males		Females		
	N	%	N	%	p	N	%	N	%	p	N	%	N	%	p	N	%	N	%	p
Apples	26	78.8	18	66.7	0.291	20	51.3	18	66.7	0.214	19	59.4	20	66.7	0.553	7[1]	100.0	8	80.0	0.208
Grapes	20	60.6	17	63.0	0.852	26	66.7	20	74.1	0.520	15	46.9	17	56.7	0.441	6	85.7	5	50.0	0.129
Apricots	24	72.7	18	66.7	0.610	28	71.8	18	66.7	0.656	15	46.9	18	60.0	0.301	5	71.4	7	70.0	0.949
Peaches	24	72.7	17	63.0	0.419	26	66.7	16	59.3	0.539	16	50.0	21	70.0	0.109	5	71.4	7	70.0	0.949

Nectarines	15	45.5	13	48.1	0.835	16	41.0	18	66.7	0.040	10	31.3	14	46.7	0.213	3	42.9	8	80.0	0.115
Strawberries	21	63.6	20	74.1	0.387	19	48.7	14	51.9	0.802	17	53.1	15	50.0	0.806	4	57.1	8	80.0	0.309
Cucumbers	25	75.8	21	77.8	0.854	26	66.7	20	74.1	0.520	23	71.9	20	66.7	0.657	5	71.4	7	70.0	0.949
Celery	5	15.2	6	22.2	0.481	5	12.8	5	18.5	0.526	7	21.9	9	30.0	0.465	1	14.3	5	50.0	0.129
Cherry tomatoes	7	21.2	7	25.9	0.668	14	35.9	6	22.2	0.235	8	25.0	8	26.7	0.881	1	14.3	5	50.0	0.129
Peas	3	9.1	4	14.8	0.429	7	17.9	2	7.4	0.220	2	6.3	0[1]	0.0	0.164	0[1]	0.0	2	20.0	0.208
Spinach	4	12.1	4	14.8	0.760	4	10.3	5	18.5	0.336	5	15.6	4	13.3	0.798	0[1]	0.0	3	30.0	0.110
Tomatoes	29	87.9	24	88.9	0.903	34	87.2	23	85.2	0.816	29	90.6	27	90.0	0.934	7[1]	100.0	10[1]	100.0	NA
Potatoes	30	90.9	22	81.5	0.285	34	87.2	21	77.8	0.314	26	81.3	27	90.0	0.328	6	85.7	10[1]	100.0	0.218
Peppers	27	81.8	24	88.9	0.445	28	71.8	21	77.8	0.585	23	71.9	26	86.7	0.153	5	71.4	10[1]	100.0	0.072
Green, leafy vegetables	19	57.6	16	59.3	0.895	19	48.7	14	51.9	0.802	16	50.0	17	56.7	0.599	3	42.9	9	90.0	0.060
Tuna	3	9.1	1	3.7	0.4005	3	7.7	3	11.1	0.635	1	3.1	1	3.3	0.963	0[1]	0.0	0[1]	0.0	NA
Salmon	0[1]	0.0	1	3.7	0.265	0[1]	0.0	0[1]	0.0	NA	0[1]	0.0	0[1]	0.0	NA	0[1]	0.0	0[1]	0.0	NA
Sardine	5	15.2	1	3.7	0.141	3	7.7	2	7.4	0.966	2	6.3	0[1]	0.0	0.164	1	14.3	0[1]	0.0	0.218
Mackerel	0[1]	0.0	0[1]	0.0	NA	0[1]	0.0	1	3.7	0.223	1	3.1	0[1]	0.0	0.329	0[1]	0.0	1	10.0	0.388
Hake	1	3.0	0[1]	0.0	0.362	1	2.6	1	3.7	0.791	1	3.1	3	10.0	0.271	0[1]	0.0	1	10.0	0.388
Herring	0[1]	0.0	0[1]	0.0	NA	0[1]	0.0	0[1]	0.0	NA	0[1]	0.0	0[1]	0.0	NA	0[1]	0.0	0[1]	0.0	NA
Catfish	0[1]	0.0	2	7.4	0.112	0[1]	0.0	0[1]	0.0	NA	0[1]	0.0	0[1]	0.0	NA	0[1]	0.0	0[1]	0.0	NA
Carp	1	3.0	2	7.4	0.439	0[1]	0.0	2	7.4	0.084	1	3.1	1	3.3	0.963	1	14.3	0[1]	0.0	0.218
Sterlet	0[1]	0.0	0[1]	0.0	NA	0[1]	0.0	0[1]	0.0	NA	0[1]	0.0	0[1]	0.0	NA	0[1]	0.0	0[1]	0.0	NA

[1] This category is not used in comparisons because its column proportion is equal to zero or one.

Table S37. Exposure to BPA through different sources of the enrolled patients with SCLC and NSCLC based on their gender (given as proportions of the exposed respondents)

	SCLC					NSCLC				
	Males		Females			Males		Females		
	N	%	N	%	p	N	%	N	%	p
Bagged instant soups	16	48.5	13	48.1	0.979	32	41.0	27	40.3	0.929
Canned foods	22	66.7	9	33.3	0.010	37	47.4	23	34.3	0.110
Heating up foods in plastic containers	3	9.1	2	7.4	0.814	4	5.1	3	4.5	0.855

Table S38. Exposure to BPA through different sources of the enrolled lung cancer patients based on the diagnosed cancer type (given as proportions of the exposed respondents)

	SCLC		Lung adenocarcinoma		Squamous cell lung cancer		Neuroendocrine lung cancer		
	N	%	N	%	N	%	N	%	p
Bagged instant soups	29	48.3	29	43.9	22	35.5	8	47.1	0.519
Canned foods	31	51.7	32	48.5	20	32.3	8	47.1	0.140
Heating up foods in plastic containers	5	8.3	4	6.1	3	4.8	0[1]	0.0	0.753

1 This category is not used in comparisons because its column proportion is equal to zero or one.

Table S39. Exposure to BPA through different sources of the enrolled lung cancer patients according to the diagnosed cancer type and their gender (given as proportions of the exposed respondents)

	SCLC					Lung adenocarcinoma					Squamous cell lung cancer					Neuroendocrine lung cancer				
	Males		Females			Males		Females			Males		Females			Males		Females		
	N	%	N	%	p	N	%	N	%	p	N	%	N	%	p	N	%	N	%	p
Bagged instant soups	16	48.5	13	48.1	0.979	18	46.2	11	40.7	0.663	11	34.4	11	36.7	0.851	3	42.9	5	50.0	0.772
Canned foods	22	66.7	9	33.3	0.010	19	48.7	13	48.1	0.964	13	40.6	7	23.3	0.146	5	71.4	3	30.0	0.092
Heating up foods in plastic containers	3	9.1	2	7.4	0.814	2	5.1	2	7.4	0.703	2	6.3	1	3.3	0.593	0[1]	0.0	0[1]	0.0	NA

[1] This category is not used in comparisons because its column proportion is equal to zero or one.

Table S40. Exposure to polytetrafluoroethylene of the enrolled patients with SCLC and NSCLC according to their gender (given as proportions of the exposed respondents)

	SCLC					NSCLC				
	Males		Females			Males		Females		
	N	%	N	%	p	N	%	N	%	p
Exposure to polytetrafluoroethylene	24	72.7	13	48.1	0.051	50	64.1	45	67.2	0.699

Table S41. Exposure to polytetrafluoroethylene of the enrolled lung cancer patients based on the diagnosed cancer type (given as proportions of the exposed respondents)

	SCLC		Lung adenocarcinoma		Squamous cell lung cancer		Neuroendocrine lung cancer		
	N	%	N	%	N	%	N	%	p
Exposure to polytetrafluoroethylene	37	61.7	45	68.2	39	62.9	11	64.7	0.881

Table S42. Exposure to polytetrafluoroethylene of the enrolled lung cancer patients according to the diagnosed cancer type and their gender (given as proportions of the exposed respondents)

	SCLC					Lung adenocarcinoma					Squamous cell lung cancer					Neuroendocrine lung cancer				
	Males		Females			Males		Females			Males		Females			Males		Females		
	N	%	N	%	p	N	%	N	%	p	N	%	N	%	p	N	%	N	%	p
Exposure to polytetrafluoroethylene	24	72.7	13	48.1	0.051	27	69.2	18	66.7	0.826	18	56.3	21	70.0	0.263	5	71.4	6	60.0	0.627

Table S43. Proportions of the enrolled patients with SCLC and NSCLC who reported to have amalgam dental fillings based on their gender

	SCLC					NSCLC				
	Males		Females			Males		Females		
	N	%	N	%	p	N	%	N	%	p
Amalgam dental fillings	20	60.6	17	63.0	0.852	34	43.6	32	47.8	0.615

Table S44. Proportions of the enrolled lung cancer patients with amalgam dental fillings according to the diagnosed cancer type

	SCLC		Lung adenocarcinoma		Squamous cell lung cancer		Neuroendocrine lung cancer		
	N	%	N	%	N	%	N	%	p
Amalgam dental fillings	37	61.7	31	47.0	27	43.5	8	47.1	0.204

Table S45. Proportions of the enrolled lung cancer patients with amalgam dental fillings according to the diagnosed cancer type and their gender

	SCLC					Lung adenocarcinoma					Squamous cell lung cancer					Neuroendocrine lung cancer				
	Males		Females			Males		Females			Males		Females			Males		Females		
	N	%	N	%	p	N	%	N	%	p	N	%	N	%	p	N	%	N	%	p
Amalgam dental fillings	20	60.6	17	63.0	0.852	17	43.6	14	51.9	0.508	13	40.6	14	46.7	0.632	4	57.1	4	40.0	0.637
